# Translation in Giant Viruses: A Unique Mixture of Bacterial and Eukaryotic Termination Schemes

**DOI:** 10.1371/journal.pgen.1003122

**Published:** 2012-12-13

**Authors:** Sandra Jeudy, Chantal Abergel, Jean-Michel Claverie, Matthieu Legendre

**Affiliations:** CNRS, Aix-Marseille Université, IGS UMR7256, Marseille, France; Fred Hutchinson Cancer Research Center, United States of America

## Abstract

Mimivirus and Megavirus are the best characterized representatives of an expanding new family of giant viruses infecting Acanthamoeba. Their most distinctive features, megabase-sized genomes carried in particles of size comparable to that of small bacteria, fill the gap between the viral and cellular worlds. These giant viruses are also uniquely equipped with genes coding for central components of the translation apparatus. The presence of those genes, thought to be hallmarks of cellular organisms, revived fundamental interrogations on the evolutionary origin of these viruses and the link they might have with the emergence of eukaryotes. In this work, we focused on the Mimivirus-encoded translation termination factor gene, the detailed primary structure of which was elucidated using computational and experimental approaches. We demonstrated that the translation of this protein proceeds through two internal stop codons via two distinct recoding events: a frameshift and a readthrough, the combined occurrence of which is unique to these viruses. Unexpectedly, the viral gene carries an autoregulatory mechanism exclusively encountered in bacterial termination factors, though the viral sequence is related to the eukaryotic/archaeal class-I release factors. This finding is a hint that the virally-encoded translation functions may not be strictly redundant with the one provided by the host. Lastly, the perplexing occurrence of a bacterial-like regulatory mechanism in a eukaryotic/archaeal homologous gene is yet another oddity brought about by the study of giant viruses.

## Author Summary

Giant viruses, such as Mimivirus and Megavirus, have huge near-micron-sized particles and possess more genes than several cellular organisms. Furthermore their genomes encode functions not supposed to be in a virus, such as components of the protein translation apparatus. Since Lwoff in 1957, viruses are defined as ultimate obligate intracellular parasites from their need to hijack the peptide synthesis machinery of their host to replicate. We looked at the Mimivirus and Megavirus proteins that recognize the stop codons, the translation termination factors. We found that these genes contain two internal stop codons, meaning that their translation bypasses two distinct stop codons to produce a functional translation termination factor. These types of autoregulatory mechanisms are found in bacterial termination factors, although it involves only a single internal stop codon and not two, and are absent from their eukaryotic and archaeal homologs. Despite these bacterial-like features, giant viruses' termination factors have sequences that do not resemble bacterial genes but are clearly related to the eukaryotic and archaeal termination factors. Thus, giant viruses' termination factors surprisingly combine elements from eukaryotes/archaea and bacteria.

## Introduction

The first giant virus, Mimivirus, was discovered ten years ago [1]. This double stranded DNA virus infecting amoebae of the *Acanthamoeba* genus exhibits a record-breaking particle more than 700 nm in diameter and a 1.2 Mb genome, larger than several cellular genomes [2]. Remarkably this virus possesses 1018 genes [3], i.e. twice the number of genes found in the bacteria *Buchnera aphidicola* [4], the archaea *Nanoarchaeum equitans* [5] or the eukaryotic endosymbiotically derived *Hemiselmis andersenii* nucleomorph [6]. Importantly several genes of this giant virus encode functions previously thought to be hallmarks of the cellular world, the most striking being central components of the translation machinery. For instance the Mimivirus genome encodes 4 different aminoacyl-tRNA synthetases that specifically attach amino acids to their cognate tRNAs. Transcriptome analyses showed that these genes are expressed in a regulated manner during the viral replication cycle [7], thus making them unlikely to be pseudogenes. Moreover functional and structural studies of the Mimivirus Methionyl- and Tyrosyl-tRNA synthetases proved that they are genuine functional enzymes [8]. However, the loop involved in the recognition of the tRNA anticodon by the Tyrosyl-tRNA synthetase is shorter in Mimivirus, suggesting that only two bases are recognized rather than three in the cellular enzymes [8]. So not only do giant viruses' genomes encode unexpected genes, but these genes are clearly different from their known cellular counterparts, ruling out a simple horizontal gene transfer (HGT). Collectively these elements fuelled the debate on the origin of giant viruses, on their living or nonliving condition [9], [10], and whether they belong to a 4^th^ domain of life as some authors even claimed [11], [12].

Two main scenarios can explain the presence of cell-specific genes in a virus. On the one hand this can be due to massive horizontal gene transfers between the host (or its intracellular parasites) and the virus [13]. On the other hand this could be the result of the reductive evolution of an ancient more complex cellular ancestor [14]. Our recent discovery of Megavirus, a new giant virus relative of Mimivirus shed some light on these fundamental issues. Megavirus has a larger capsid, longer genome and wider gene content than Mimivirus or any other characterized virus to date [15]. Importantly, all the Mimivirus genes involved in translation have an ortholog in Megavirus. Furthermore three additional aminoacyl-tRNA synthetases were found in this new giant virus. It then becomes very unlikely that the translation-related genes found in the Mimivirus and Megavirus genomes were acquired by HGT. A more parsimonious scenario is simply that these genes were already present in the common ancestor of Mimivirus and Megavirus, leading to the hypothesis that this ancestor was endowed with an even more complete translation apparatus, inherited from an ancestral cellular organism [12], [16]. We reasoned that further studying other giant virus-encoded translation components might provide additional insights on the nature of this ancestor.

Translation of messenger RNAs into proteins is a complex and multistep process. It involves three major stages: initiation, elongation and termination. It is noteworthy that Mimivirus and Megavirus encode 5 orthologous genes, in addition to the aminoacyl-tRNA synthetases, that are involved in these three phases [2], [15]. This suggests that a tight control of the translation process is required for the optimal progress of the virus replication cycle, and that the virally-encoded factors function in a way that cannot be assumed by their cellular counterparts. Each of the above steps is essential for optimal protein synthesis. Accurate termination for instance allows correct decoding of the mRNA, as well as promotes the proper dissociation and recycling of the ribosomes. Two functional classes of release factors (RFs) mediate translation termination (summarized in Table S1). The class-I RF recognizes the stop codon located in the ribosomal A-site and then releases the polypeptide chain, assisted by the class-II GTPase RF. There are two class-I RFs in bacteria, RF1 which recognizes UAA/UAG stop codons, and RF2 which recognizes UAA/UGA stop codons. In eukaryotes and archaea, there is a single omnipotent class-I RF called eRF1 and aRF1 respectively, capable of recognizing all three stop codons. Whereas eRF1 and aRF1 share conserved sequence motifs and are functionally and structurally related, they are highly divergent in sequence and structure from the bacterial RF1/RF2 [17], [18], with the exception of a uniquely conserved GGQ motif. The class-II GTPase RFs, called RF3 in bacteria and eRF3 in eukaryotes are also unrelated, and do not exhibit sequence similarity apart from their GTPase domain [19]. In addition, eRF3 is an essential gene in eukaryotes while RF3 is lacking in some bacterial lineages [20]. They also function differently: whereas eRF3 and eRF1 physically interact to release the peptide [21], RF3 interacts with the ribosome to remove RF1/RF2 from the A site [22]. Finally, although the eukaryotic eRF1 and the archaeal aRF1 class-I RFs are closely related, no obvious eRF3 class-II ortholog could be found in archaeal genomes. This has been puzzling for a long time until the discovery that the omnipotent archaeal elongation factor 1 α (aEF1α) is able to bind aRF1 and functions as a class-II RF [23], [24]. In summary, although the function of RFs is as universal as the stop codons, the proteins involved in the termination of translation are clearly different between bacteria on the one hand, and eukaryotes and archaea on the other.

Translation termination is globally highly accurate but occasionally leads to unfaithful decoding of the gene sequence. Mis-terminations of polypeptide, the so-called translational recoding events, are of two types: the “stop codon readthroughs” and the frameshifts [25]. Readthroughs are caused by the binding of an aminoacyl-tRNA in lieu of a release factor when the ribosome encounters the stop codon. This leads to translation proceeding in the same frame upstream and downstream of the stop codon. A near-cognate tRNA such as the glutamine tRNA (close to the UAG stop codon) or the tryptophan tRNA (close to the UGA stop codon) can be incorporated [26]. Alternatively a cognate but non-standard tRNA can be involved, for instance the tRNA suppressors [27] and the selenocysteine tRNAs [28]. The other type of error, translational frameshift, is caused by a leap of one or two nucleotides leading to the pursuit of translation, albeit in a different reading frame. The occurrence of such mis-terminations can be programmed to act as a powerful regulatory mechanism. One of the most elegant genetic switches involves a programmed translational frameshift in the bacterial RF2 class-I RF [29]. In 70% of surveyed bacteria, RF2 appears to be composed of two partially overlapping open reading frames (ORFs) [30]. The first ORF terminates by a UGA stop codon, immediately followed by a second ORF (in a +1 frame) encoding the rest of the protein. When functional RF2 is plentiful, a high proportion of ribosomes terminates at UGA to synthesize a short non-functional N-terminal RF2 peptide. Since full-length RF2 is then in limited amount, the normal processing of the UGA stop codon (peptide release) is stalled, enhancing the probability of a frame shift, and thereby favoring the translation of a complete functional RF2 protein. This negative feedback loop, exclusively found in the bacterial RF, can thus buffer RF2 concentration and enable subtle controlling of translation termination [29], [31].

In this study, we started from the discovery that the class-I translation RFs homologs present in the Mimivirus (R726 gene) and Megavirus (mg280 gene) giant viruses had been wrongly annotated. We then established the correct structure of these genes by predicting a unique combination of two recoding events: a readthrough and a frameshift, shared by both viral genes. Further computational analyses as well as several lines of experimental evidences validated the new gene structure and the recoding events, which can thus act as autoregulatory elements. Unexpectedly, these viral class-I RF homologs uniquely combine regulatory features specific to the bacterial domain with a clear sequence resemblance with class-I RFs of the eukaryotic and archaeal types. Once again this raises the question of the origin and evolution of the translation components found in giant viruses.

## Results

### Gene structure of the Mimivirus/Megavirus peptide chain release factors

Mimivirus R726 gene is annotated as a class-I peptide chain release factor. According to previously published transcriptomic data [7] its 5′UTR is 640 nt long, which makes it the longest 5′UTR among the 979 Mimivirus protein-coding genes. R726 5′UTR length is 20.5 standard deviations above the average of 12.5 nt. This anomalous 5′UTR length prompted us to reexamine the initial annotation of R726.

**Figure 1:**
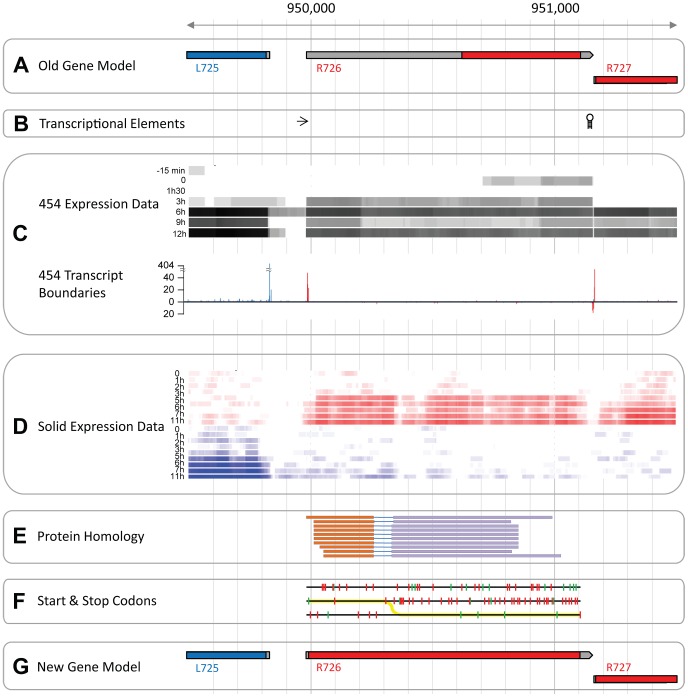
The Mimivirus R726 gene re-annotation. A) Initial genes models are shown. Coding sequences (CDSs) in the forward strand are colored in red and those in the reverse strand in blue. The untranslated regions (UTRs) are in grey. B) The arrow depicts the early transcription promoter element [66] and the hairpin symbol stands for the Mimivirus transcription termination signal [67]. C) RNA-seq data from the 454 sequencing technology (from [7]). The heatmap in the upper part of the panel shows the coverage of the RNA-seq reads once mapped to the genome. Highly covered genomic positions are in black and uncovered positions in white. Each row corresponds to a different time-point of the transcriptome experiment, from the earliest time-point (top), to the end of the infection cycle (bottom). The graph in the lower part of the panel shows the number of RNA-seq reads starting or ending at a given position. Only reads corresponding to cDNAs with a 5′-end specific tag are shown above the x-axis, and reads with a 3′-end specific tag are shown below the x-axis. Peaks of reads matching the forward strand are in red and the ones matching the reverse strand in blue. D) RNA-seq data from the SOLiD sequencing technology (from [3]). Same as C, except that the coverage of reads is strand-specific with forward strand coverage colored in red and reverse strand in blue. E) Genomic regions with protein sequence similarity. Each line corresponds to a matching protein and each color corresponds to a particular frame. F) Each line corresponds to one of the 3 forward strand frames. Red ticks show the potential start codons and green ticks the stop codons. The yellow line depicts the most parsimonious path to decode the protein. G) Same as A but with genes models inferred from this study.

Predictions of unusually large 5′ UTR (see Figure 1A) most often arise from mistakes in the definition of the transcript boundaries, in this case however, several elements argue against such an explanation. First, known transcriptional regulatory elements flank the predicted transcript while none were found inside it (Figure 1B). Furthermore, our 454 RNA-seq data (from [7]) covered the entire R726 transcript and thus supported the annotation (see Figure 1C). The incorporation of 5′ and 3′ specific tags at the extremities of the cDNAs allowed us to precisely map transcriptional start sites (TSS) and transcriptional end sites (TES) (see [7] for details). Figure 1C again shows that R726 TSS and TES coincide with the annotated transcript boundaries. Finally, an independent and strand-specific dataset from total RNA sequenced on the SOLiD plateform (from [3]) confirmed the transcript boundaries as well (Figure 1D). Altogether these results indicate that the R726 transcript annotation is correct.

A second possibility is that the abnormally long 5′UTR arose from an error in the prediction of the R726 protein sequence. For instance, an upstream methionine codon could constitute the actual translation initiation. We explored this possibility by searching for R726 homologous sequences in the UniProtKB/Swiss-Prot database using the blast program and the R726 genomic sequence as a query. The 10 best matching proteins (with an E-value<1e^−27^) are shown in Figure 1E. Two findings emerged from this test. First, the sequence similarity at the protein level was clearly not restricted to the annotated coding region but covered the entire R726 transcript sequence. Second, the alignments of the matching proteins were systematically split between two alternative frames. This suggested that the actual R726 coding region started upstream of the bioinformatic prediction and involved a frameshift. Potential start and stop codons in the three frames are shown in Figure 1F, as well as the most parsimonious path to encode a protein more fully homologous to the other release factors. This resulted into a new gene model (see Figure 1G) encoding a full-length protein via two recoding events: a readthrough of the first encountered stop codon in the 5′ ORF, and a frameshift at the next downstream stop codon.

To eliminate the trivial possibility that these two stop codons were due to errors in the R726 gene sequence, we first re-sequenced the R726 genomic region using traditional Sanger sequencing. In addition we exploited our very high coverage SOLiD re-sequencing of Mimivirus genomic DNA (from [3]). The R726 genomic sequence was found to be identical in both cases (Figure S1), including the predicted readthrough and frameshift stop codons.

We then examined the more remote possibility that the mRNA sequence could differ from the genomic sequence following RNA editing. For this we first sequenced R726 cDNAs using Sanger sequencing (see Figure S1). In addition, we mapped the RNA-seq data from two independent experiments from polyadenylated [7] and total RNA [3] to the R726 genomic region. Figure S1 clearly shows that the R726 transcript sequence is identical to the genomic sequence. Therefore the two stop codons must be present at the mRNA level.

**Figure 2:**
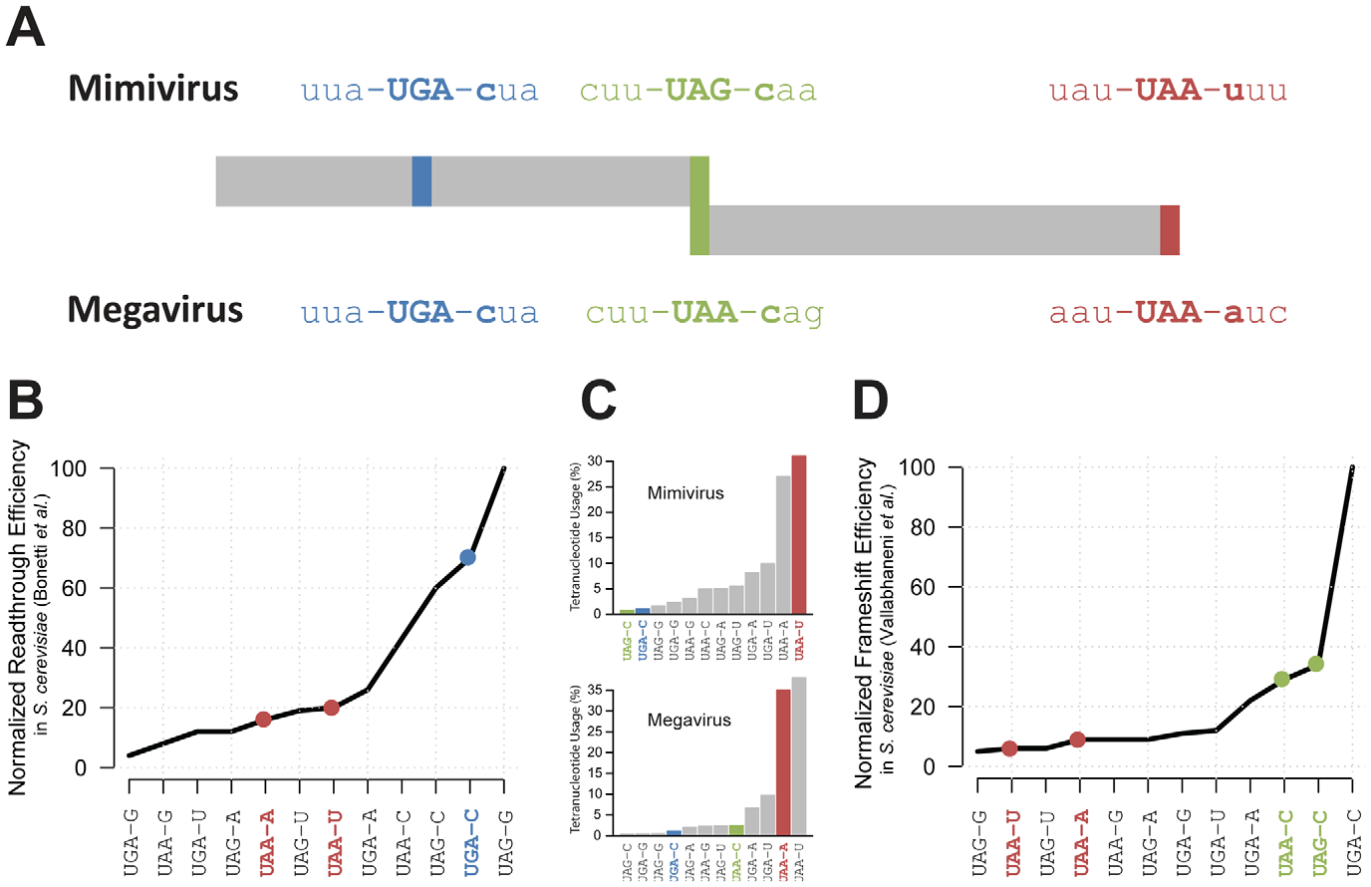
Representation of Mimivirus R726 and Megavirus mg280 coding regions. A) Representation of Mimivirus R726 and Megavirus mg280 coding regions. The sequences of the three stop codons and their neighboring codons are shown. The readthrough stop is in blue, the frameshift stop in green and the last stop in red. B) The readthrough efficiency in *S. cerevisiae* of all possible stop codons tetranucleotides is reported from [32]. The values were normalized as a percentage of the most efficient tetranucleotide. C) Histogram of the percentage of stop codons tetranucleotides used in Mimivirus (top) and Megavirus (bottom). The three stops colors correspond to A. D) The frameshift efficiency in *S. cerevisiae* of all possible stop codons tetranucleotides is reported from [36]. The values were normalized as a percentage of the most efficient tetranucleotide.

An alternative explanation for the odd R726 coding sequence could be that the Mimivirus gene is a pseudogene. However, the two previously described RNA-seq datasets (from [7] and [3]) consistently ranked R726 as one of the most expressed Mimivirus genes during the replication cycle. Indeed R726 is in the highest quartile of total gene expression (Figure S2). Furthermore, the R726 ortholog in the Megavirus genome (mg280) presents exactly the same gene structure pattern (see Figure 2A), that is first a readthrough followed by a downstream frameshift in the 5′ region of the gene. It is worth noting that the readthrough stop codon (UGA) is strictly conserved between the two viruses, while the Mimivirus UAG frameshift stop codon is substituted by a UAA stop codon in Megavirus. Once reconstructed, the full-length protein sequence from Mimivirus (R726) and Megavirus (mg280) exhibited 47% of identical residues, a percentage comparable to the average sequence similarity of the Mimivirus/Megavirus orthologous protein pairs [15]. The fact that the stop codons and the recoding pattern are conserved between R726 and mg280 despite their level of sequence divergence, strongly suggests that they are translated as predicted here and function as proteins. Furthermore, as Mimivirus and Megavirus only share 50% of their genes [15], it would be unlikely for these two orthologous ORFs to be conserved if they were in fact pseudogenes.

### A readthrough recoding event in the Mimivirus class-I RF

According to our hypothesis, the production of a functional R726 protein requires translation to occasionally proceed beyond the readthrough stop. We thus examined whether this stop codon was likely to be read through. A crucial factor for readthrough to occur is not the stop codon sequence *per se* (UAA, UAG or UGA) but rather the sequence context around it. For instance the first nucleotide downstream of the stop codon is known to be the strongest determinant of readthrough efficiency [32]. We thus compared the tetranucleotides composing Mimivirus and Megavirus readthrough stop codons with available experimental data of readthrough efficiency measurements in eukaryotes (*S. cerevisiae* in [32]). As shown in Figure 2A and Figure 2B, the UGA-C tetranucleotide of Mimivirus and Megavirus readthrough stop codons is very efficiently read through, i.e. it is a weak terminator. Conversely the two tetranucleotides encompassing the genuine 3′ stop codons (UAA-U in Mimivirus and UAA-A in Megavirus) are not favorable to readthrough. If UAA-U and UAA-A really efficiently terminate polypeptide chains while UGA-C promotes frequent readthrough in giant viruses, the Mimivirus and Megavirus stop codons should exhibit a tetranucleotide usage reflecting this bias. As expected, Figure 2C shows that the tetranucleotides of R726 and mg280 genuine stops are among the most frequently used whereas the tetranucleotide of the readthrough stop is very rarely used.

**Figure 3:**
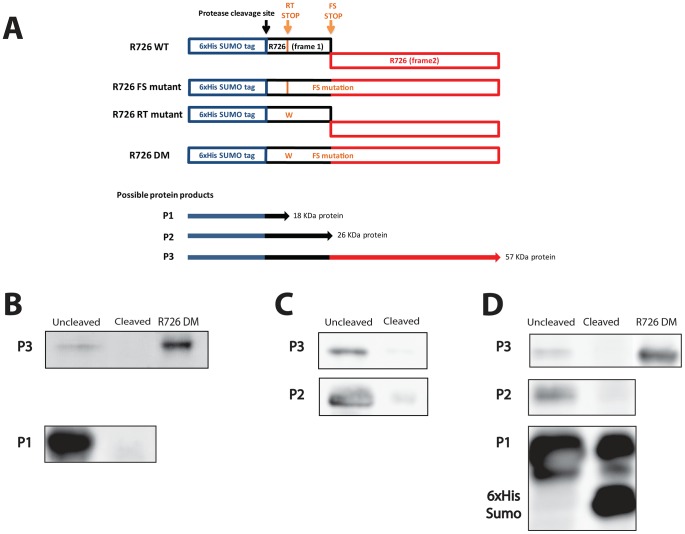
Schematic representation of the Mimivirus R726 gene constructs and Western blots. A) Schematic representation of the Mimivirus R726 gene constructs. The R726 WT construct is the wild-type gene in frame with a cleavable 6×His-SUMO tag (blue). The two R726 frames are symbolized by black and red boxes. The position of the Prescission cleavage site is depicted with a black arrow. The readthrough stop (RT stop) and the frameshift stop (FS stop) are symbolized by orange arrows. The R726 FS mutant construct exhibits a mutation at the FS stop to create a +1 translational frameshift. The RT stop in the R726 RT mutant construct was mutated by tryptophan substitution (see main text). The R726 DM double mutant construct exhibits both mutations to produce a full-length R726 protein. P1, P2 and P3 correspond to the three possibly expressed proteins from the constructs. The western blots show the expression of B) the P1 and P3 proteins from the R726 FS mutant construct, C) the P2 and P3 proteins from the R726 RT mutant construct and D) the P1, P2 and P3 proteins from the R726 WT construct. The P2 and P3 proteins are not detectable after cleavage of the tag with Prescission protease. Due to the large quantity of P1 protein, a fraction remains uncleaved after protease digestion and is still visible on the gel. The R726 DM gene product was used as a positive control for full-length R726 protein expression. Entire gels are shown in Figure S3.

We then went on the experimental confirmation that the Mimivirus R726 first stop can be read through. Since no usable system for protein expression in the Mimivirus host (*Acanthamoeba castellanii*) is currently available, we used *Escherichia coli* as expression host. We reasoned that the occurrence of such recoding events in this organism makes the demonstration possible [33]. Furthermore, the strength of the termination in *E. coli* depends on sequences that are similar to the ones in eukaryotes [32], [34]–[36]. We thus first cloned the full-length gene, i.e. containing the readthrough stop and the frameshift stop, into a modified pET vector in frame with an N-terminal 6×His-SUMO tag (Figure 3A, R726 WT construct). We then performed site-directed mutagenesis to get rid of the frameshift stop by removing the first nucleotide of the UAG stop codon to create a +1 translational frameshift. The resulting construct (R726 FS mutant) corresponds to the R726 gene containing only the readthrough stop, in frame with a 6×His-SUMO tag (Figure 3A). The R726 FS mutant was then transformed in *E. coli* for protein expression. The proteins were purified by Nickel affinity chromatography and the elution fraction was analyzed by western blot using antibodies raised against the 6×His tag of the potentially produced proteins. The western blot revealed two bands running around 20 KDa and 60 KDa (Figure 3B and Figure S3A), possibly corresponding to the expected protein products from the R726 FS construct: a short peptide ending at the readthrough stop (Figure 3A, P1) and a full-length protein product resulting from the readthrough of this first stop codon (Figure 3A, P3). We incubated the elution fraction with the Prescission protease which should cleave the two products if they include the 6×His-SUMO tag. As expected, the 20 KDa and the 60 KDa proteins were no longer detected after cleavage showing that they correspond to the P1 and P3 predicted R726 gene products. In addition, the double mutant construct lacking the two stop codons (Figure 3A, R726 DM construct) corresponding to the R726 full-length product and the 60 KDa product migrate at the same position on the gel. These results demonstrate that in *E. coli* readthrough can occur at the first stop of the Mimivirus R726 gene.

We then investigated which amino-acid was incorporated at the first R726 stop codon. In some organisms the UGA stop codon, such as the R726 and mg280 readthrough stops, leads to the incorporation of a selenocysteine (Sec). We failed to identify Sec tRNAs in the Mimivirus and Megavirus genomes. However, we found that the *A. castellanii* genome encodes a highly expressed Sec tRNA (see Figure S4 and Figure S5). Similarly, the protein machinery required for Sec insertion is lacking from the Mimivirus and Megavirus genomes but is present in *A. castellanii* (see Table S2). Finally, we looked for genes targeted by the Sec incorporation machinery based on the presence of a specific Sec insertion sequence (SECIS) element. SECIS elements were indeed found in the 3′UTR of *A. castellanii* genes encoding homologs to known selenoproteins (see Table S3), and correlating with the presence of UGA stop codons. By contrast, SECIS elements were found neither in Mimivirus genes encoding homologs to known selenoproteins nor in the R726 gene. Taken together these results suggest that selenocysteine incorporation occurs in *A. castellanii* but not in Mimivirus.

As no cognate tRNA decodes the first R726 stop codon we searched for natural near-cognate tRNAs. Among the only two types of tRNAs shared by Mimivirus and Megavirus (leucine and tryptophan), tryptophan tRNAs (Trp-tRNAs) was previously shown to recognize UGA stop codons [37], [38]. Furthermore, Mimivirus Trp-tRNA is one of the most expressed tRNAs from the Mimivirus/*A. castellanii* system (see Figure S5). Interestingly, Mimivirus (and Megavirus) Trp-tRNA exhibits an adenine in the D arm that is similar to the mutation in the well-studied Hirsh suppressor (see Figure S6) [39]. This *E. coli* tRNA derived from a Trp-tRNA recognizes UGA stop codons through a G-to-A mutation in the D arm. Given these congruent elements, we hypothesized that tryptophan is the most likely amino acid to be incorporated at the readthrough stop in Mimivirus and Megavirus class-I RFs.

### A frameshift recoding event in the Mimivirus class-I RF

We predict that once the ribosome proceeds beyond the readthrough stop, a frameshift should occur at the downstream stop to produce a functional class-I RF in Mimivirus and Megavirus. Similarly to readthrough recoding events, the frequency of ribosomal frameshifting is highly dependent on the surrounding sequences. Again, it has been shown that the first base downstream of the stop codon is correlated with the frequency of frameshifting [34]. Therefore, we compared the tetranucleotides at both Mimivirus and Megavirus frameshift stops with experimentally determined translational frameshifting efficiency in eukaryotes (*S. cerevisiae* from [36]). Figure 2A and Figure 2D show that the Mimivirus frameshift stop tetranucleotide (UAG-C) and the Megavirus one (UAA-C) are amongst the most efficient frameshifting inducers. By contrast, frameshifting frequency is low at the genuine stops (UAA-U in Mimivirus and UAA-A in Megavirus). The tetranucleotide usage in Mimivirus genes stop codons strengthens this observation. As shown in Figure 2C, UAG-C is used at a rate of less than 1% as a translation termination signal in Mimivirus, whereas UAA-U is the most frequently used tetranucleotide (more than 30%). We observed the same trend in Megavirus (Figure 2C). In addition the full “CUU UAG C” motif in Mimivirus and “CUU UAA C” in Megavirus are similar to the conserved “CUU UGA C” shifting motif found in the bacterial RF2 programmed frameshift [30]. Collectively these results support the occurrence of frameshifting recoding events in R726 and mg280.

To experimentally address whether the second stop codon in R726 is prone to frameshifting, we performed site-directed mutagenesis on the wild-type gene to get rid of the readthrough stop. The R726 readthrough stop was thus replaced by a tryptophan, resulting in a construct containing a 6×His-SUMO tag in frame with the 5′ part of the R726 gene (Figure 3A, R726 RT mutant). There are two protein products expected from this construct: a small protein that ends at the frameshift stop (Figure 3A, P2) and a full-length protein resulting from a frameshift recoding event at this locus (Figure 3A, P3). The plasmid was transformed in *E. coli* for protein expression. The proteins were then purified by Nickel affinity chromatography and the elution fraction was analyzed by SDS-PAGE and western blotting. The western blot revealed the two expected bands, one corresponding to a 25–30 KDa protein and a second band around 60 KDa (Figure 3C and Figure S3B). We thus incubated the elution fraction with the Prescission protease and, as expected, the two bands disappeared, supporting that they correspond to the P2 and P3 protein products, respectively. Moreover, the 60 KDa band was detectable on a Coomassie blue stained gel (Figure S3B), which allowed us to analyze it by mass spectrometry. We demonstrated without ambiguity (E-value = 9.4e^−17^) that it corresponded to the full-length 6×His-SUMO R726 protein. The identified peptides covered 58% of the full-length protein, from its N-terminal to its extreme C-terminal (Figure S7). This result clearly shows that +1 translational frameshifting can occur at the R726 second stop in *E. coli*.

At this point we experimentally demonstrated that translation can proceed beyond the two stop codons independently (the readthrough stop and the frameshift stop). Finally, the wild-type gene was expressed to verify whether its translation would result in the predicted full-length R726 protein. The purified product was analyzed by western blot (Figure 3D and Figure S3C) and revealed the three expected bands: one highly expressed of 20 KDa, one in the 25–30 KDa range and the 60 KDa full-length protein. Prescission digest of the purified fraction showed that the three bands correspond to the P1, P2 and P3 protein products, respectively. Altogether these results demonstrate that a full-length R726 protein can be produced from the wild-type Mimivirus gene.

### A new type of class-I RF

We showed that the R726 Mimivirus gene is able to bypass its two internal stop codons and produce a full-length protein, although it remains to be verified whether this protein is a genuine peptide chain release factor. Homology searches using the blast program against the UniProtKB/Swiss-Prot database identified class-I RFs from eukaryotes (best E-value = 8e^−25^) and archaea (best E-value = 1e^−25^) as the best matches to the R726 protein sequence. In contrast, no significant match was detected with any of the bacterial RFs (neither RF1 nor RF2).

**Figure 4:**
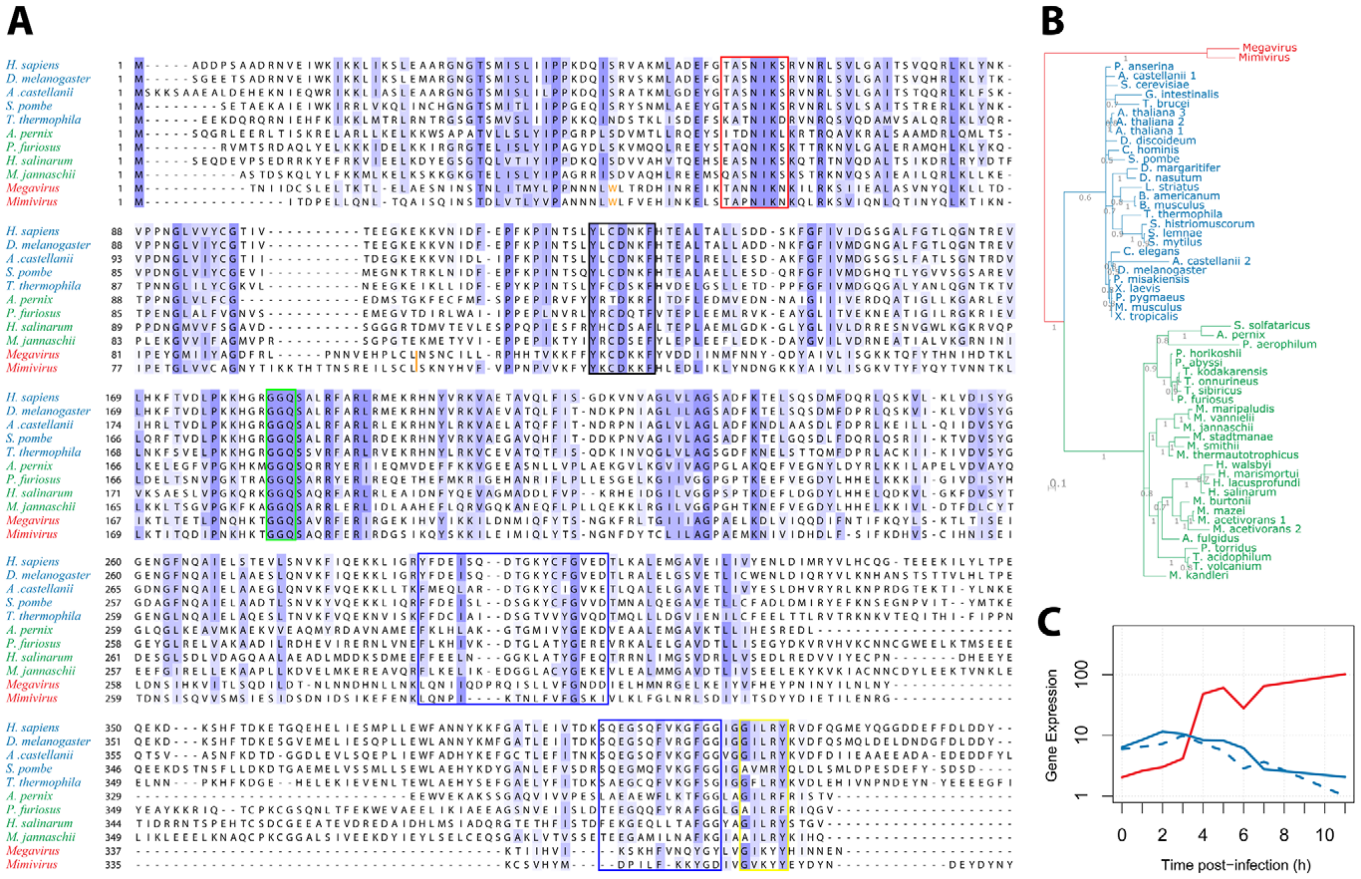
Multiple alignment of Mimivirus R726 and Megavirus mg280. A) Multiple alignment of Mimivirus R726 and Megavirus mg280 protein sequences (in red), as well as representative sequences from eukaryotic eRF1 (in blue) and archaeal aRF1 proteins (in green). Functionally important regions are boxed (see main text for a detailed description). The orange tryptophan is at the readthrough stop in Mimivirus and Megavirus sequences (see main text). The orange bar depicts the position of the Mimivirus and Megavirus frameshift stops. B) Phylogeny of Mimivirus, Megavirus, eukaryotic and archaeal class-I RFs using a Bayesian analysis of 58 sequences of 750 amino acid positions (321 ungapped) under the CAT60 mixture model (see materials and methods for details). The colors are the ones used in A. Branch support shown represents posterior probabilities and bar represents 0.1 substitutions per site. C) Gene expression profile (using RNA-seq data from [3]) of Mimivirus R726 gene is shown in red, the solid blue line shows the *A. castellanii* eRF1 expression and the dashed blue line shows the *A. castellanii* paralog expression.

We then examined the R726 sequence for the presence of key functional elements previously described in the eRF1/aRF1 peptide chain release factors. Figure 4A displays a multiple alignment of R726 and mg280 with representative sequences from eukaryotes and archaea class-I RFs. First, this alignment shows that the giant viruses and the eukaryote/archaea proteins are globally well conserved. Two conserved regions in the N-terminal part of the class-I RFs are well-known to be involved in the recognition of the stop codon. Those are the (TAS)NIKS motif (Figure 4A, red box) [40] and the YxCxxxF motif (black box) [41]. These crucial elements are conserved in the Mimivirus and Megavirus homologs. In addition, the peptidyl-tRNA hydrolysase activity of the class-I RFs requires a universally conserved GGQ motif in the middle of the protein [17]. Again, this essential motif is present in the Mimivirus and Megavirus homologs (Figure 4A, green box). The interaction of class-I RF with class-II RF (in eukaryotes) or aEF1α (in archaea), involves amino acids located in the C-terminal part of eRF1/aRF1. The blue boxes (Figure 4A) highlight the regions of known interacting residues in eukaryotes [21] and archaea [24]. The GILRY motif (Figure 4A, yellow box) is also known to mediate the interaction between eRF1 and eRF3 [42]. These regions, although less conserved than the N-terminal part of the protein, also exhibit residues that are found in Mimivirus and Megavirus as well. In contrast, none of the essential functional motifs present in the bacterial class-I RFs (see [43] for review) are found in R726 and mg280, with the exception of the GGQ motif. We can thus conclude that R726 has all the sequence hallmarks of a genuine class-I RF of the eukaryotic/archaeal type.

Even though eRF1, aRF1 and R726/mg280 are globally well conserved, the giant viruses' RFs exhibit specific elements (Figure 4A). For instance there is an insertion in the N-terminal part of the protein, as well as a large deletion in the C-terminal domain, partially overlapping a previously identified deletion in *Aeropyrum pernix* [24]. Mimivirus and Megavirus sequences are clearly the most divergent sequences of the alignment. This visual impression was objectively confirmed by reconstructing the phylogeny of these class-I RFs, using the Phylobayes software with the CAT mixture model [44]. This method was used as it is known to better fit the phylogenetic signal present in giant viruses' genes than traditional evolutionary models [45]. The tree in Figure 4B exhibits a tight grouping of the eukaryotic sequences within one branch, a tight grouping of the archaeal sequences within a second branch, and a third branch consisting of the Mimivirus and Megavirus homologs. Other Bayesian and maximum likelihood methods supported the same three-pronged tree topology with the exception of a deeper branching of an *A. castellanii* paralog (Figure S8). This paralog does not contain internal stop codons similarly to the other eukaryotic class-I RFs. Hence R726 and mg280 are representative sequences of a new type of class-I RF.

We previously showed that the R726 transcript was strongly expressed. The timing of its expression and the interplay with host's genes is illustrated in Figure 4C. Both *A. castellanii* genes, the canonical eRF1 and the paralog, see their expression slowly decreasing along the viral replication cycle. In contrast, the expression of the Mimivirus homolog clearly raises in an opposite manner. This negative correlation suggests that the expression of the Mimivirus class-I RF compensates for the decline of the host RF.

## Discussion

An apparent anomaly in the annotation of the predicted Mimivirus class-I release factor homolog led us to investigate in more details its transcript structure. This resulted in the hypothesis that Mimivirus possesses an intricate translation termination process involving the recoding of two stop codons. A similar gene structure in Megavirus strengthens this prediction that was then verified experimentally. To our knowledge such a combined occurrence of a frameshift and a readthrough in the coding sequence of a class-I RF has never been reported in any lineage in the tree of life. Surprisingly, although the sequences of the Mimivirus and Megavirus class-I RF homologs show close proximity with the eukaryotic/archaeal peptide chains release factors, they incorporate an autoregulatory mechanism only found in bacterial class-I RFs. As a central component of the translation apparatus, RFs are not found in viruses with the exception of the two recently described unclassified nucleocytoplasmic large DNA viruses: Marseillevirus [46] and Lausannevirus [47]. However these genes do not contain internal stop codons and are likely recent HGT from their cellular host (see Figure S9).

An increasing number of studies support the idea that giant viruses have ancient origins, possibly predating the radiation of eukaryotes [2], [11], [12], [16], [48], [49]. The phylogenetic reconstruction of the Mimivirus and Megavirus RFs genes, deeply branching at the root of eukaryotes and archaea, is consistent with this view (see Figure 4B). Furthermore since Mimivirus/Megavirus RFs bear no clear phylogenetic affinity with any extant cellular homolog, acquisition by recent HGT is very unlikely. Thus, as for other translation components found in giant viruses, the Mimivirus and Megavirus RFs could originate from an ancestral genome encoding a complete translation system [8], [15], [16].

According to the current dogma, eukaryotes derived from the archaeal/bacterial domains, therefore one can hypothesize that the giant viruses' release factors regulatory mechanism could have been inherited from their prokaryotic ancestor. This is consistent with the fact that only bacterial RFs are known to exhibit a shifting motif analogous to the one we detected in Mimivirus/Megavirus RFs. Furthermore the only identified recoding event in Mimivirus and Megavirus corresponds to the RF gene, out of the more than 1000 genes encoded by each viral genome. Finally, this unusual recoding event is surprisingly present in the functional homolog to one of the rare bacterial gene exhibiting the same regulatory trick. It is thus tempting to speculate that the cenancestor possessed this regulatory element that was kept in the bacterial and Mimivirus/Megavirus lineages, but lost in the other lineages (eukaryotes and archaea). Nevertheless, this scenario is impossible to prove in the apparent absence of sufficient sequence/structural similarity between the bacterial RF genes and the eukaryotic/archaeal RF genes [17], [18].

The alternative hypothesis involves the reinvention of a similar regulatory feature in the giant viruses' lineage. This would be a nice example of convergent evolution that could have occurred before the divergence of Mimivirus and Megavirus. The multiple invention of the termination factor frameshifting mechanism in different bacterial lineages has been proposed previously [30]. Finally, the regulatory mechanism might also have been present in the ancestor of giant viruses, archaea and eukaryotes but subsequently lost in the two cellular lineages, and perhaps substituted by other more complex regulatory mechanisms.

Effective translation termination requires the interaction of the class-I RF with a GTPase class-II RF (eRF3) in eukaryotes, or a GTPase elongation factor (aEF1α) in archaea, through the C-terminal domain [23], [24]. We showed that the Mimivirus R726 and Megavirus mg280 genes are likely to be class-I RFs of the eukaryotic/archaeal type although they constitute a new separate clade (Figure 4B). They could thus also interact with a translational GTPase, among which the host's eRF3 is a candidate. Such a subtle host-pathogen interaction should be supported by an enhanced similarity of the viral C-terminal class-I RF with the host protein. This is clearly not the case (see Figure 4A), which makes this interaction uncertain. Alternatively the giant viruses could encode their own class-II RF, making them autonomous for the translation termination function. There is no evidence of such class-II RF homologs in Mimivirus and Megavirus genomes, but the interacting protein could be one of the numerous genes of unknown function shared by the two viruses [15]. Another possibility would be that the giant viruses follow the archaeal model and recruit a pluripotent translation GTPase factor [24] encoded in their genome. The Mimivirus R624 gene could be this pluripotent interacting partner as it is annotated as a translation elongation factor, and it shares significant sequence similarity with the eukaryotic eRF3 and the archaeal aEF1α (best E-values<1e^−10^) proteins referenced in the trGTPbase (http://www.GTPbase.org.uk). However R624 was shown to be related to the GBP-1 subfamily of GTPases [11], which is consistent with our phylogenetic reconstruction (Figure S10). The function of GBP-1 is still vague, but it seems to be related to protein synthesis [50] and mRNA surveillance [51]. Finally, one cannot rule out the possibility that the giant viruses' class-I RFs have no class-II RFs interacting partners as is the case in many groups of bacteria [20], which would further highlight the hybrid bacterial/eukaryotic nature of giant viruses RFs. This last hypothesis is reinforced by previous studies reporting that mutations in the TASNIKS stop codon recognition motif abolish the eRF3 requirement for peptide release at the UAA and UAG stop codons [31], [52]. Since Mimivirus and Megavirus contain motifs that are not strictly identical to this consensus motif, the class-II RF might thus be dispensable for translation termination.

The programmed frameshift in the bacterial RF2 induces an autoregulatory feedback loop that maintains a constant production of termination factor [31]. It has been proposed that such a mechanism primarily aims to prevent excessive RF2 protein concentration which limits false recognition of tryptophan UGG codons as stops [31]. The two internal stop codons in giant viruses' RFs likely induce an even stronger buffering of protein overexpression. The R726 transcript expression appears to compensate for the host class-I RF expression decline, at least during the late phase of infection (Figure 4C). Translation termination function might thus rely on the viral enzyme, and its tight regulation at the translation step is needed to maintain a low yet constant amount of viral termination factor. The strong regulation might be a way to control viral genes that contain stop codons prone to frequent translational frameshifts and readthroughs (Figure 2) and thus produce alternative protein variants. The RF concentration leverage would then directly regulate their final product length. However, we did not find evidence for such regulated genes in the Mimivirus and Megavirus genomes. Beyond this speculative hypothesis, it is clear that the virally-encoded RFs are not strictly functionally redundant to the one provided by the host. Future experimental studies will help to understand how giant viruses rely on their own encoded translation factors, as well as the functional role of such a complex system for translation termination regulation.

In addition to their enormous particle and genome size, and the presence of numerous translation components [2], [15], the unique combined occurrence of both a frameshift and a readthrough in a translation termination factor is yet another oddity brought about by the study of giant viruses.

## Materials and Methods

### *A. castellanii* genome assembly and annotation

The *A. castellanii* genome assembly (available at http://www.hgsc.bcm.tmc.edu/microbial-detail.xsp?project_id=163) is composed of 54,947 contigs (18,936 scaffolds). We used this basis to perform a complete re-assembly of the genome using all available sequence data. We gathered *A. castellanii* genomic DNA sequences from the NCBI trace archive. The complete dataset was composed of 689,389 Sanger reads and 10,556,721 454 reads. We performed a hybrid assembly using the Arachne [53] and Phrap (P. Green, http://www.phrap.org) assemblers. We finally obtained a 44 Mb *A. castellanii* genome assembly composed of 549 contigs (ranging from 3,412 nt to 1,183,386 nt) with a N50 of 17,363 nt. We subsequently performed the genome annotation using the Augustus gene prediction algorithm [54] incorporating gene expression data and protein homology evidences. The complete proteome of *Dictyostelium pupureum* and *Dictyostelium discoideum*, as well as the UniProtKB/Swiss-Prot database, were aligned to the *A. castellanii* genome using exonerate with the protein2genome model [55]. The same program was also used with the est2genome model to map all available *A. castellanii* ESTs from [7], from http://www.hgsc.bcm.tmc.edu/microbial-detail.xsp?project_id=163 and from Genbank, to the *A. castellanii* genome. All together these data allowed Augustus to predict 14,343 protein-coding genes. A total of 491 tRNAs was also predicted using the tRNAscan-SE program [56].

### Selenocysteine analyses

Proteins homologous to known selenoproteins and components of the selenocysteine incorporation machinery were searched using the HMMer program (http://www.hmmer.org) with HMM profiles from [57], against the *A. castellanii* and Mimivirus proteomes. SECIS elements were searched using the SECISearch program [58].

### Protein multiple alignments and phylogenies

All protein multiple alignments were performed using the MAFFT algorithm [59] with the L-INS-I parameter. Phylogeny reconstructions were done using the three following methods. We used the maximum likelihood package PhyML [60] with the WAG model and 100 bootstrap replicates. We also used the MrBayes software [61] with the PhyML tree as a starting tree and a Γ distributed rate model. The algorithm was run for 1,000,000 generations, the first 2,500 of which were disregarded and trees were sampled every 100 generations. Finally the phylogeny reported in Figure 3B was performed using the PhyloBayes algorithm [62] with a C60 mixture model and a burnin parameter of 1/5 of the length of the chain. Two chains were run in parallel and the stopping criterions were: discrepancies <0.3 and effective sizes >50.

### Transcriptome analyses

454 RNA-seq sequences of Mimivirus polyadenylated RNAs were used from [7]. RNA-seq data of total RNA from the Mimivirus/*A. castellanii* system were used from [3]. The reads sequenced by the SOLiD technology were mapped to the Mimivirus and *A. castellanii* genomes using the TopHat software [63] as a first pass. We mapped the reads in color space using the following parameters: max-multihits = 1, min-intron-length = 20 and max-intron-length = 2000. We then re-aligned the unmapped reads using the Bfast software [64] in color space with a minimum normalized score of 35. Subsequently we used the Mimivirus and *A. castellanii* protein-coding and tRNA gene annotations (see above) to calculate gene expression levels. For each time point, that is 0, 1 h, 2 h, 3 h, 4 h, 5 h, 6 h, 7 h and 11 h post-infection, we converted RNA-seq exonic reads density to the standard measurement of reads per Kb per million reads (RPKM) as described in [65].

### Plasmid construction and site-directed mutagenesis

The full-length R726 gene was amplified from Mimivirus genomic DNA using specific primers flanked by SacI and NotI restriction sites. The PCR product was inserted into an in-house modified pET28 plasmid to yield a N-terminally removable His-SUMO tagged protein.

Site-directed mutagenesis of the two stop codons was performed using the QuickChange kit (Stratagene) to replace the readthrough stop by a tryptophan and/or to get rid of the frameshift stop by creating a +1 translational frameshift. The 4 plasmids containing the wild-type gene, the readthrough stop mutant, the frameshift mutant, or the double mutant, were verified by sequencing.

### Protein expression and purification

The resulting vectors were transformed into Rosetta strain (Novagen). Cells were grown into 2YT medium containing 100 µg.mL^−1^ ampicillin and 34 µg.mL^−1^ chloramphenicol at 30°C to an A_600_ of 0.9. Temperature was then shifted to 17°C for 15 minutes. The protein expression was induced by adding 0.1 mM of isopropyl β-thiogalactopyranoside. Cells were grown 16–18 h post induction. Bacteria were harvested by centrifugation and resuspended in lysis buffer containing 50 mM Tris-HCl pH 8.0, 300 mM NaCl, 10 µg.mL^−1^ DNase and EDTA-free protease inhibitor cocktail (Roche). Cells were lysed using sonication or by mechanical disruption with the FastPrep system using glass beads (MP bioscience). The crude lysate was clarified by centrifugation at 13,000× g for 45 min.

The clarified lysate was applied to a 1 ml HisTrap HP Column (GE Healthcare) charged with Ni^2+^ and equilibrated with buffer A (50 mM Tris-HCl pH 8.0, 300 mM NaCl) on an AKTÄ explorer 10S FPLC system (GE Healthcare). The column was washed with 10 column volumes of buffer A, 10 column volumes of buffer A containing 25 mM Imidazole and 20 column volumes of buffer A containing 50 mM Imidazole. Elution fraction was analyzed by SDS-PAGE and given the very low level of protein expression we used antibodies raised against the 6×His tag to reveal the recombinant proteins by western blot. For Mass spectrometry analysis, the band was cut out of the gel, trypsin digested and the resulting peptides were analyzed by MS/MS.

## Supporting Information

Figure S1 Validation of the Mimivirus R726 sequence. The Sanger re-sequencing of the region that overlaps the R726 readthrough (left dark orange column) and frameshift stops (right dark orange column) is shown with red for adenine, blue for cytosine, green for guanine and purple for thymidine. The histogram bellow shows the SOLiD DNA re-sequencing of the same region (from [3]). The reads from this NGS experiment were mapped to the genome. The percentage of A, C, G and T at each genomic position is shown using the same color code. The Sanger sequencing of the R726 cDNA is shown below, as well as two histograms of RNA-seq from a total RNA transcriptome experiment (from [3]), and a transcriptome analysis of polyadenylated RNAs (from [7]).
(PDF)

Figure S2 R726 gene expression. Gene expression from [7] (left) and [3] (right) experiments were calculated over the entire viral infection cycle. The Mimivirus genes were ranked according to their expression from the least expressed to the most expressed (X-axis). Each quartile of expression is shown in a different shade of gray. The red dot depicts the R726 gene expression.
(PDF)

Figure S3 Experimental validation of the R726 recoding events. The nomenclature of the gene constructs and protein products are the same as in Figure 3. The western blots show the expression of A) the P1 and P3 proteins from the R726 FS mutant construct (lane 1 and 2) and the P3 protein from the R726 DM construct (lane 3 and 4), B) the P2 and P3 proteins from the R726 RT mutant construct and C) the P1, P2 and P3 proteins from the R726 WT construct. The P2 and P3 proteins are not detectable anymore by the antibody after cleavage of the tag with Prescission protease. Due to the large quantity of P1 (lane 1) and P3 (lane 3) proteins, a fraction remains uncleaved after protease digestion and is still visible on the gel. It is worth noticing that the R726 full-length protein (R726 DM) used as a positive control already exhibits a wide degradation pattern. The disappearance of this profile after Prescission cleavage suggests a C-terminal degradation of the R726 protein. This degradation also applies to the other constructs. The 45 KDa band corresponds to the His-tagged Prescission protease (Presc). The P2 and P3 proteins from the expression of the R726 RT mutant construct are visible on SDS-PAGE stained with Coomassie blue, allowing the identification of the P3 protein by mass spectrometry. The most intense band (around 70 KDa) corresponds to an *E. coli* contaminant and is not detected on the western blot. The cleaved P3 product and the Prescission protease run at the same size on the gel.
(PDF)

Figure S4 Secondary structure representation of the *A. castellanii* Selenocysteine tRNA. The anticodon is highlighted in blue.
(PDF)

Figure S5 Tryptophan and selenocysteine tRNAs expression from the Mimivirus/*A. castellanii* system. The tRNAs were ranked according to their expression from the least expressed to the most expressed (left graph, X-axis). Each quartile of expression is shown in a different shade of gray. Green dots correspond to the *A. castellanii* tryptophan tRNAs and the red dot to the Mimivirus tryptophan tRNA. The blue dot depicts the expression of the *A. castellanii* selenocysteine tRNA. The summed expression of all the tryptophan tRNAs along the viral replication cycle is shown in the upper right graph (green) along with the expression of the Mimivirus tryptophan tRNA (red), while the lower right graph shows the expression of the *A. castellanii* selenocysteine tRNA.
(PDF)

Figure S6 Secondary structure representation of the Mimivirus and Megavirus tryptophan tRNAs compared to the Hirsh suppressor. Mimivirus Trp-tRNA is shown on the left, Megavirus Trp-tRNA on the right and the *Escherichia coli* Trp-tRNA (taken from [39]) in the middle. The anticodon is highlighted in blue, the G-to-A Hirsh suppressor mutation is shown in red, as well as an A-to-C suppression inducer mutation in orange. The mutation that corresponds to the *E. coli* Hirsh suppressor (see the red nucleotide) is present in Mimivirus and Megavirus tRNAs as well.
(PDF)

Figure S7 Identification by mass spectrometry of the full-length R726 protein expressed from the R726 RT mutant construct. The full-length R726 protein was identified with an E-value of 9.4e^−17^. In red are shown the trypsin digested peptides matching the sequence.
(PDF)

Figure S8 Phylogeny of R726, mg280 and cellular class-I RFs. A) The phylogenetic tree was built using MrBayes. Mimivirus and Megavirus sequences are shown in red, archaeal sequences in green and eukaryotic sequences in blue. Branch support shown represents posterior probability and bar represents 0.1 substitutions per site. B) Phylogenetic tree with the same sequences using PhyML. The bootstrap values from 100 replicates are shown (ranging from 0 to 1). The bar represents 0.1 substitutions per site.
(PDF)

Figure S9 Phylogeny of R726, mg280, Marseillevirus, Lausannevirus and cellular class-I RFs using PhyML. The bootstrap values from 100 replicates are shown (ranging from 0 to 1). Viral sequences are shown in red, archaeal sequences in green and eukaryotic sequences in blue. Branch support shown represents posterior probability and the bar represents 0.1 substitutions per site.
(PDF)

Figure S10 Phylogeny of the Mimivirus GTPase (R624), the Megavirus GTPase (mg752) and other cellular translational GTPases. The phylogenetic trees were built using MrBayes (A) and PhyML (B). Mimivirus and Megavirus sequences are shown in red, archaeal sequences in green, eukaryotic sequences in blue and bacterial sequences in purple. Each clade represents a translational GTPase subfamily. Associated functions are also shown. The bar represents 0.1 substitutions per site.
(PDF)

Table S1 Translation termination factors.
(PDF)

Table S2 Selenocysteine incorporation protein machinery in A) *A. castellanii* and B) Mimivirus.
(PDF)

Table S3 Putative selenoproteome in A) *A. castellanii* and B) Mimivirus.
(PDF)
