# Supplementary figures and images for: Translation in Giant Viruses: A Unique Mixture of Bacterial and Eukaryotic Termination Schemes
Source: PLoS Genet. 2012 Dec 13;8(12):e1003122. doi: 10.1371/journal.pgen.1003122 (PMC3521657; doi:10.1371/journal.pgen.1003122)

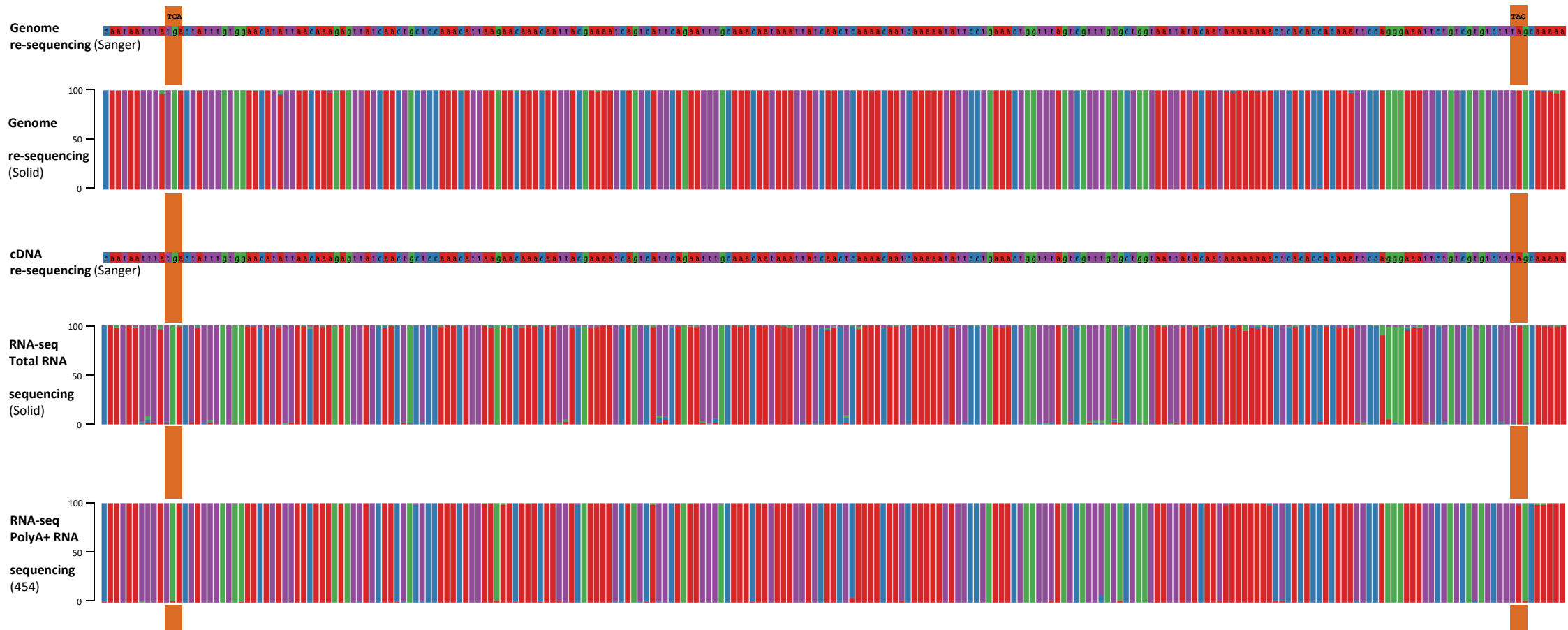

Supplement: Figure S1 — Validation of the Mimivirus R726 sequence. The Sanger re-sequencing of the region that overlaps the R726 readthrough (left dark orange column) and frameshift stops (right dark orange column) is shown with red for adenine, blue for cytosine, green for guanine and purple for thymidine. The histogram bellow shows the SOLiD DNA re-sequencing of the same region (from [3]). The reads from this NGS experiment were mapped to the genome. The percentage of A, C, G and T at each genomic position is shown using the same color code. The Sanger sequencing of the R726 cDNA is shown below, as well as two histograms of RNA-seq from a total RNA transcriptome experiment (from [3]), and a transcriptome analysis of polyadenylated RNAs (from [7]). (PDF) [file pgen.1003122.s001.pdf]

**454 Data**

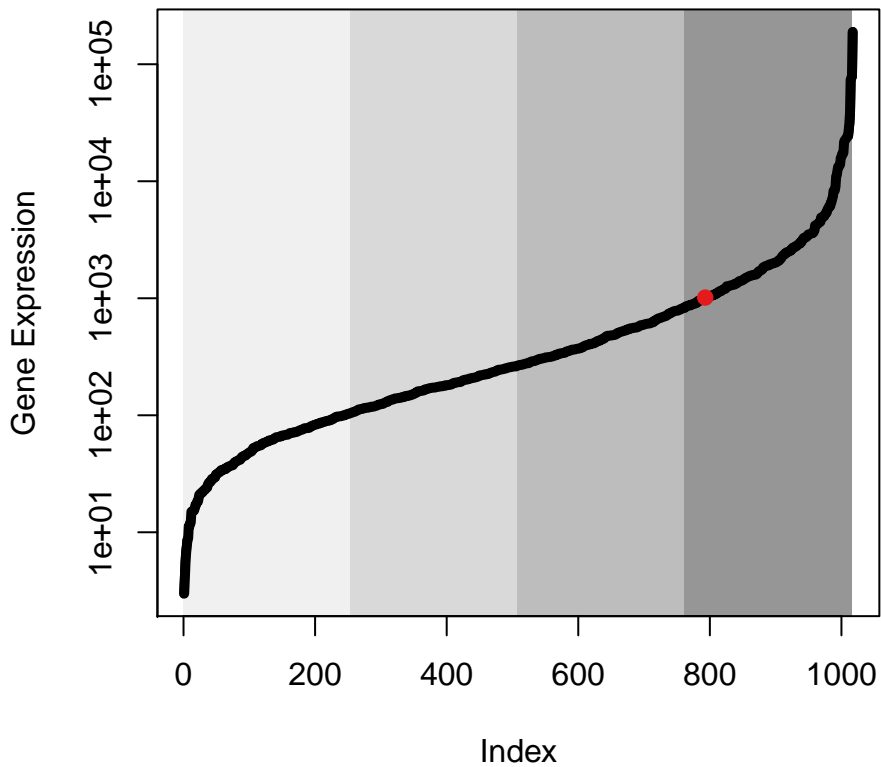

**Solid Data**

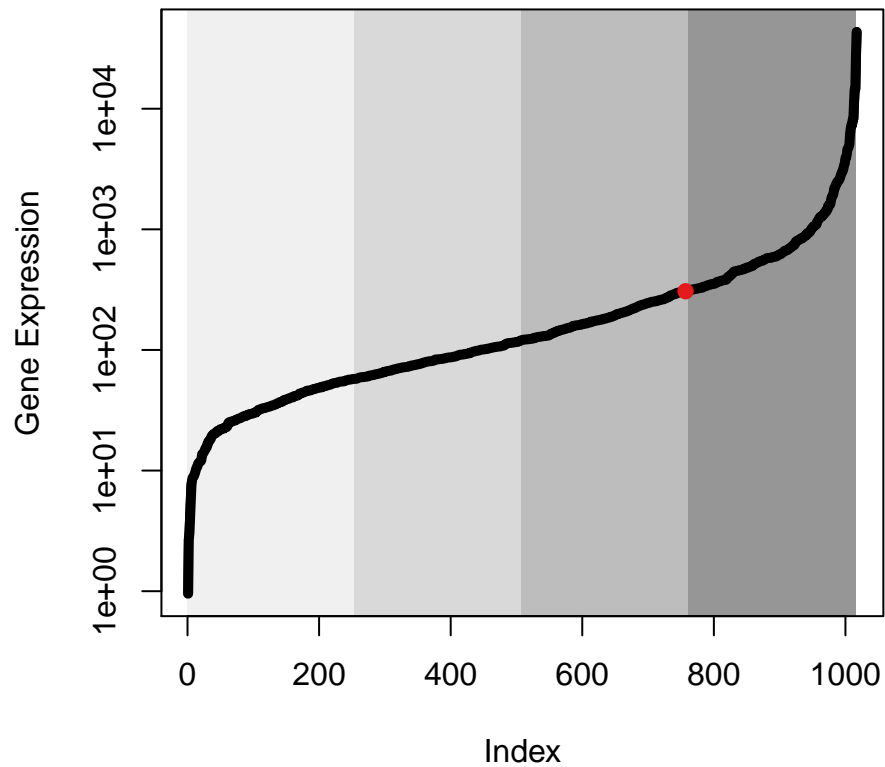

Supplement: Figure S2 — R726 gene expression. Gene expression from [7] (left) and [3] (right) experiments were calculated over the entire viral infection cycle. The Mimivirus genes were ranked according to their expression from the least expressed to the most expressed (X-axis). Each quartile of expression is shown in a different shade of gray. The red dot depicts the R726 gene expression. (PDF) [file pgen.1003122.s002.pdf]

**A**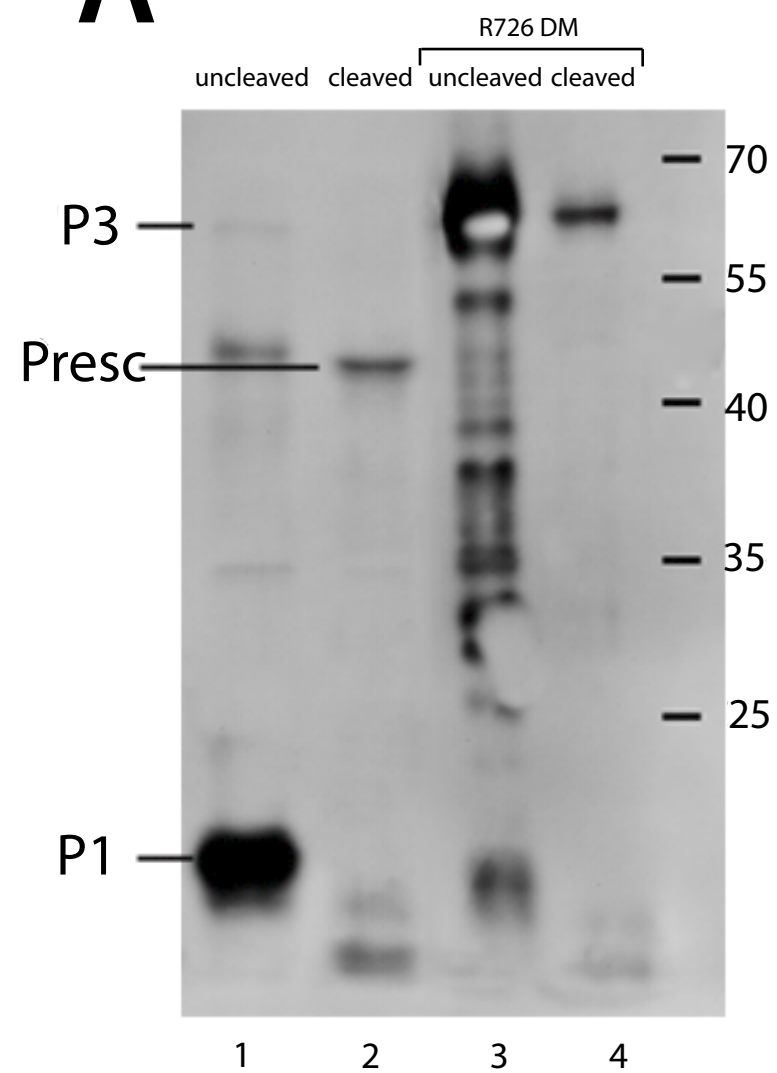**B**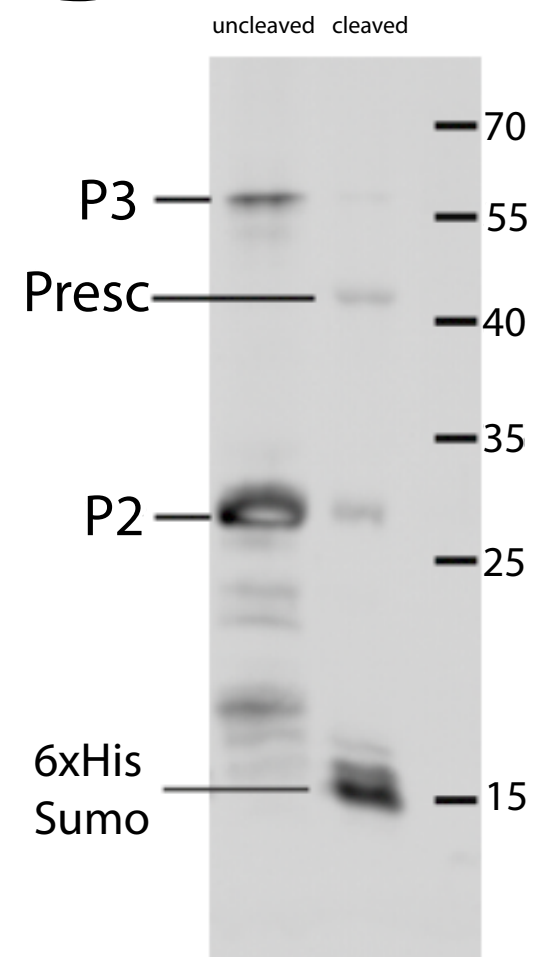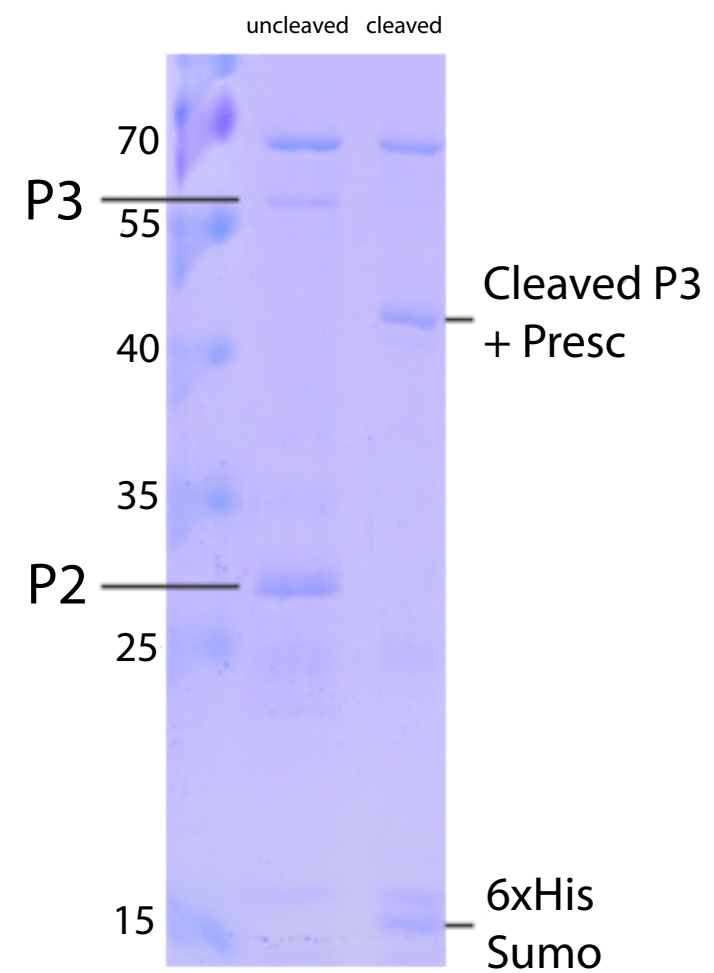**C**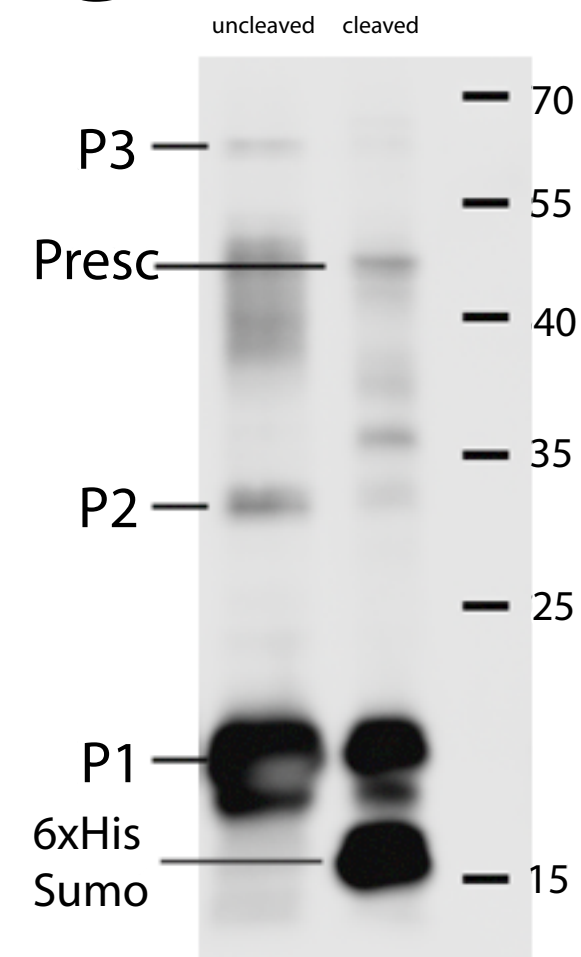

Supplement: Figure S3 — Experimental validation of the R726 recoding events. The nomenclature of the gene constructs and protein products are the same as in Figure 3. The western blots show the expression of A) the P1 and P3 proteins from the R726 FS mutant construct (lane 1 and 2) and the P3 protein from the R726 DM construct (lane 3 and 4), B) the P2 and P3 proteins from the R726 RT mutant construct and C) the P1, P2 and P3 proteins from the R726 WT construct. The P2 and P3 proteins are not detectable anymore by the antibody after cleavage of the tag with Prescission protease. Due to the large quantity of P1 (lane 1) and P3 (lane 3) proteins, a fraction remains uncleaved after protease digestion and is still visible on the gel. It is worth noticing that the R726 full-length protein (R726 DM) used as a positive control already exhibits a wide degradation pattern. The disappearance of this profile after Prescission cleavage suggests a C-terminal degradation of the R726 protein. This degradation also applies to the other constructs. The 45 KDa band corresponds to the His-tagged Prescission protease (Presc). The P2 and P3 proteins from the expression of the R726 RT mutant construct are visible on SDS-PAGE stained with Coomassie blue, allowing the identification of the P3 protein by mass spectrometry. The most intense band (around 70 KDa) corresponds to an E. coli contaminant and is not detected on the western blot. The cleaved P3 product and the Prescission protease run at the same size on the gel. (PDF) [file pgen.1003122.s003.pdf]

# *A. castellanii* Selenocysteine tRNA

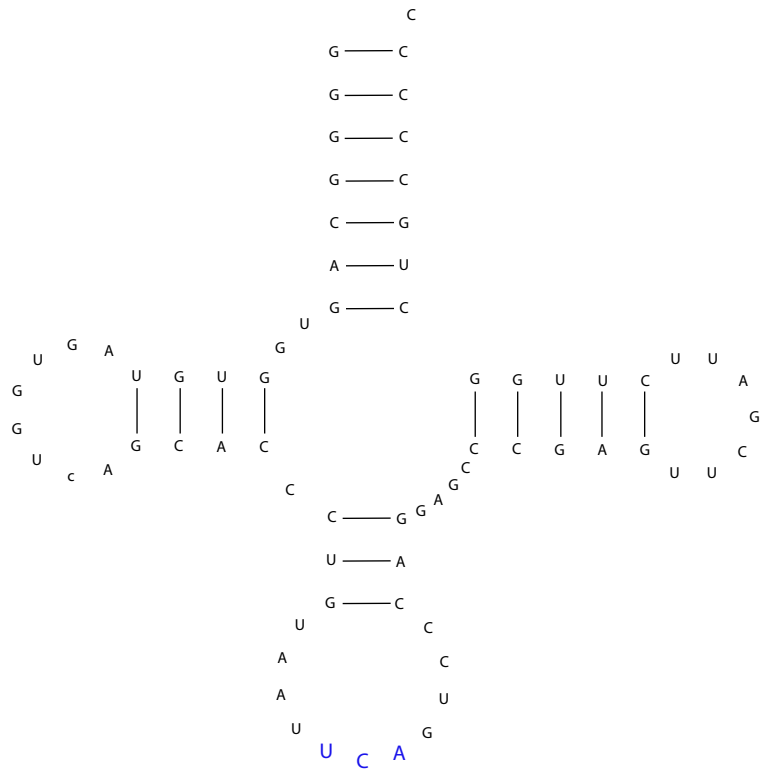

Supplement: Figure S4 — Secondary structure representation of the A. castellanii Selenocysteine tRNA. The anticodon is highlighted in blue. (PDF) [file pgen.1003122.s004.pdf]

**tRNA Expression**

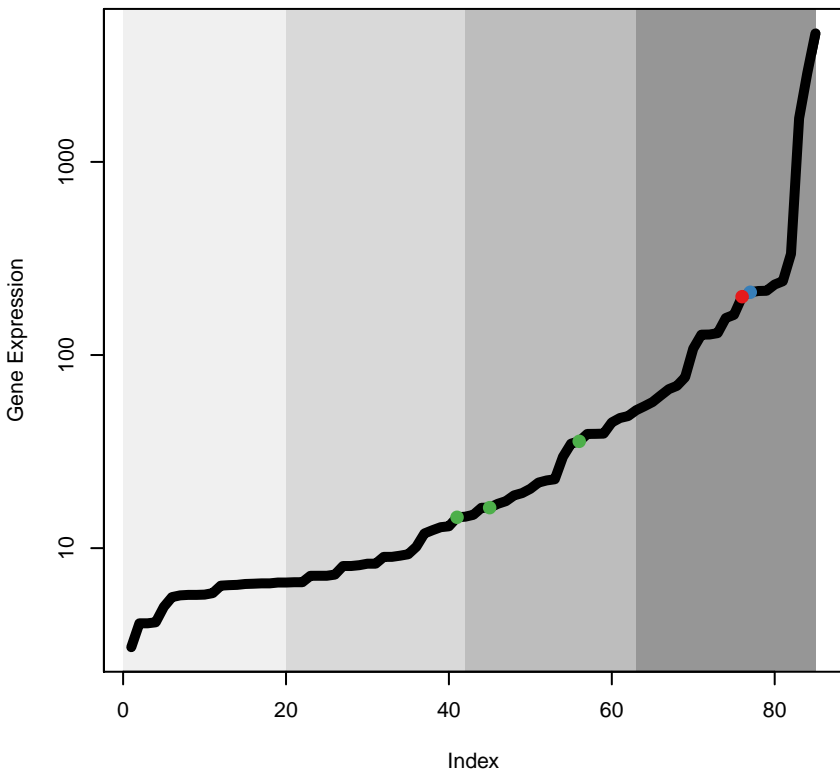

**Tryptophan tRNAs**

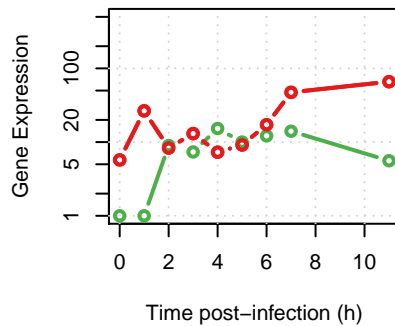

**Selenocysteine tRNA**

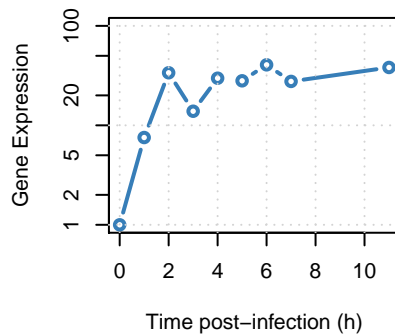

Supplement: Figure S5 — Tryptophan and selenocysteine tRNAs expression from the Mimivirus/A. castellanii system. The tRNAs were ranked according to their expression from the least expressed to the most expressed (left graph, X-axis). Each quartile of expression is shown in a different shade of gray. Green dots correspond to the A. castellanii tryptophan tRNAs and the red dot to the Mimivirus tryptophan tRNA. The blue dot depicts the expression of the A. castellanii selenocysteine tRNA. The summed expression of all the tryptophan tRNAs along the viral replication cycle is shown in the upper right graph (green) along with the expression of the Mimivirus tryptophan tRNA (red), while the lower right graph shows the expression of the A. castellanii selenocysteine tRNA. (PDF) [file pgen.1003122.s005.pdf]

# Mimivirus

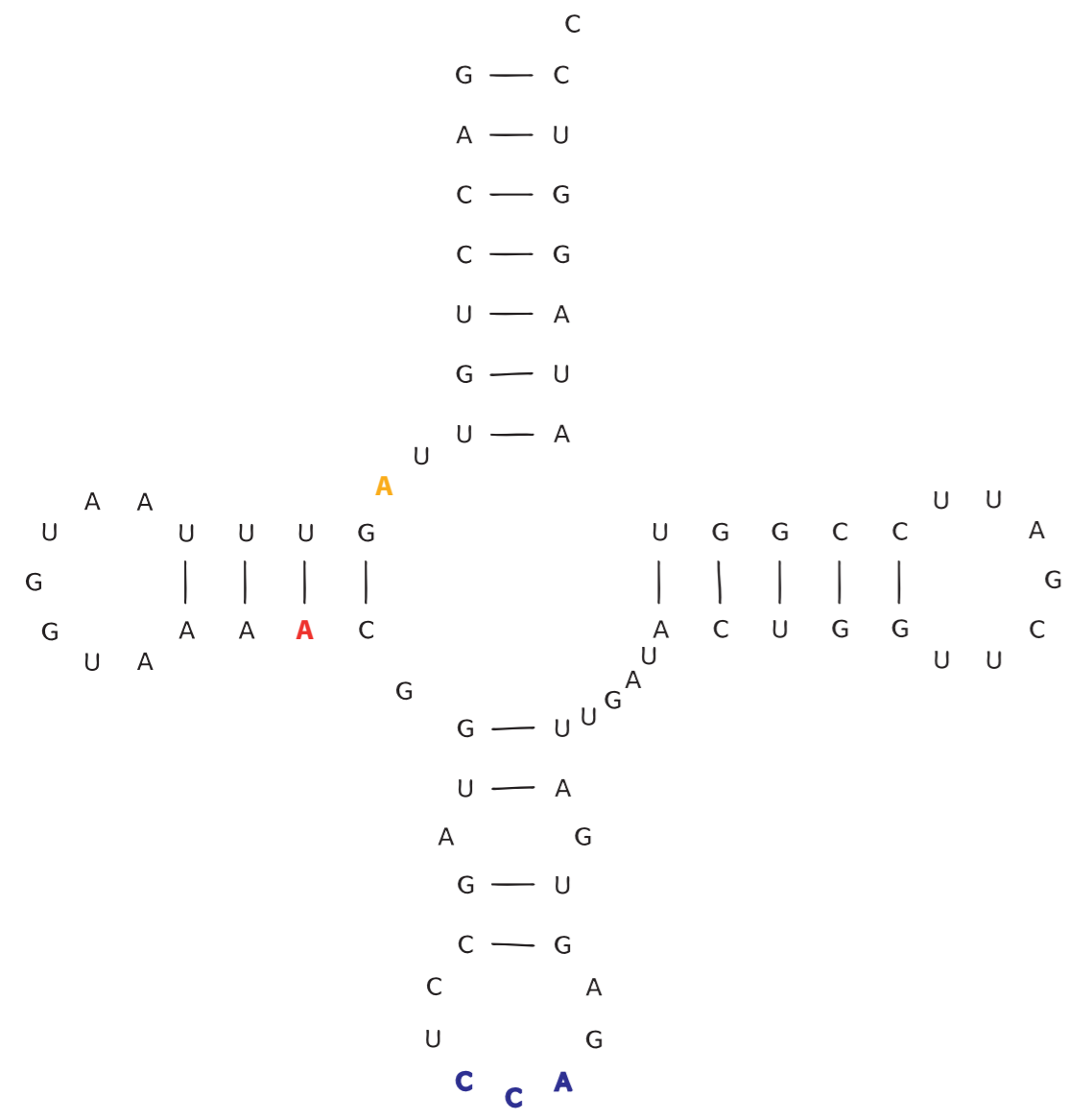

# Escherichia coli

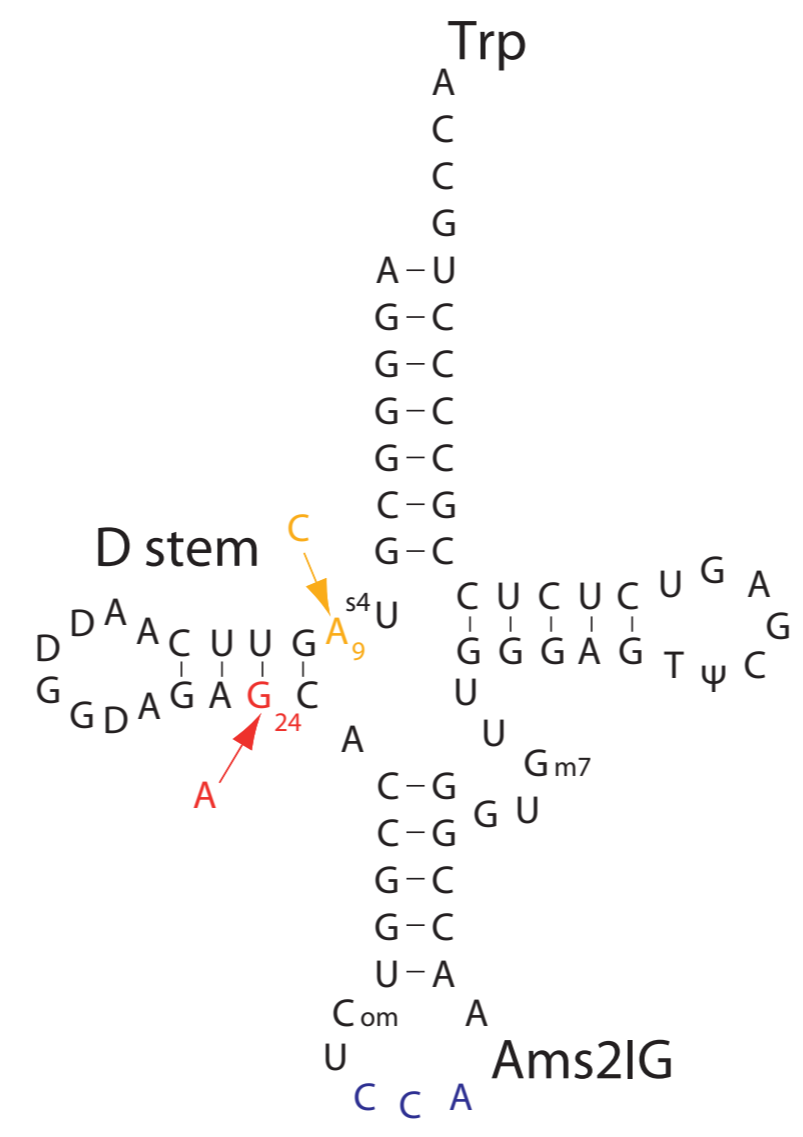

# Megavirus

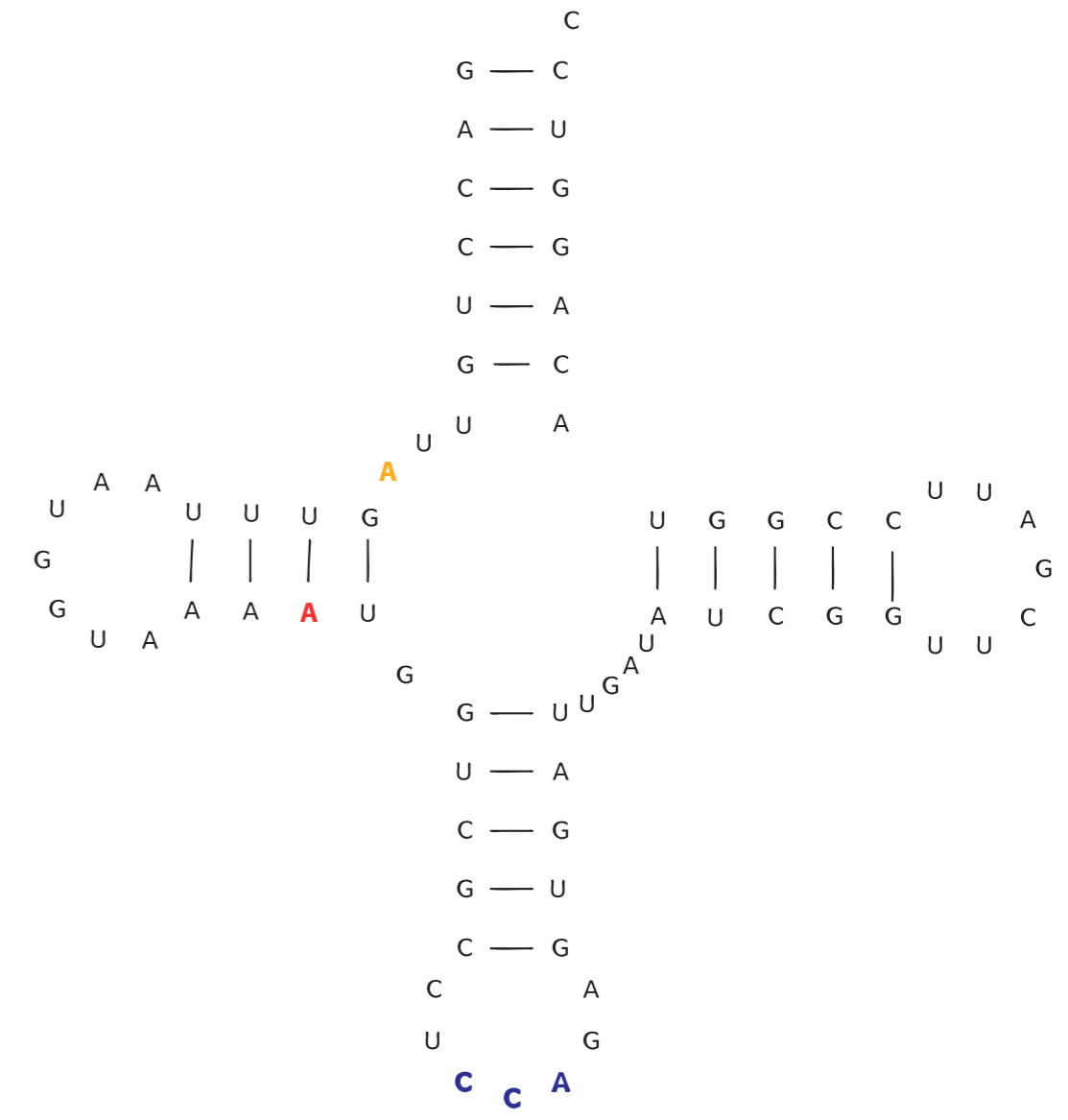

Supplement: Figure S6 — Secondary structure representation of the Mimivirus and Megavirus tryptophan tRNAs compared to the Hirsh suppressor. Mimivirus Trp-tRNA is shown on the left, Megavirus Trp-tRNA on the right and the Escherichia coli Trp-tRNA (taken from [39]) in the middle. The anticodon is highlighted in blue, the G-to-A Hirsh suppressor mutation is shown in red, as well as an A-to-C suppression inducer mutation in orange. The mutation that corresponds to the E. coli Hirsh suppressor (see the red nucleotide) is present in Mimivirus and Megavirus tRNAs as well. (PDF) [file pgen.1003122.s006.pdf]

A

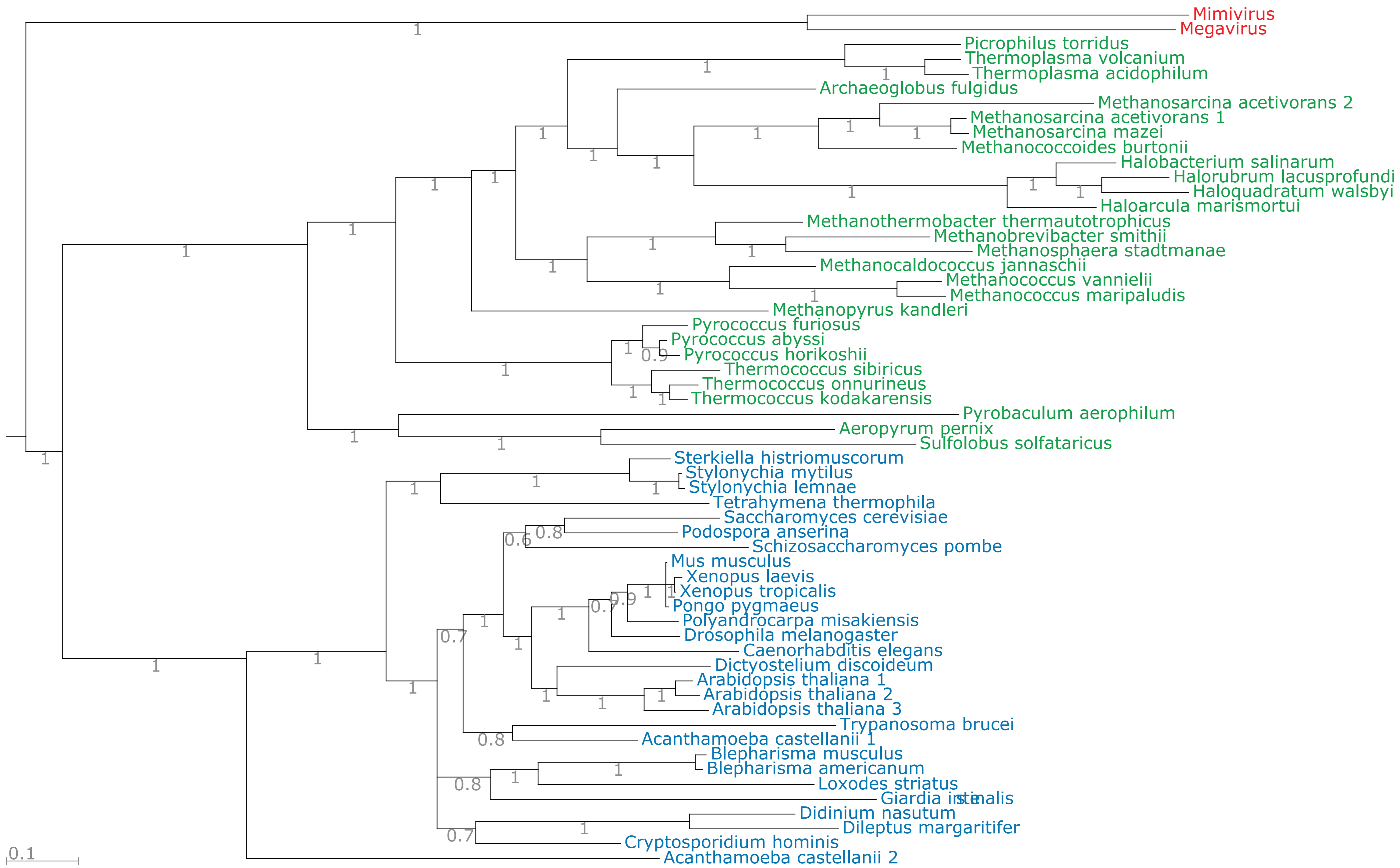

B

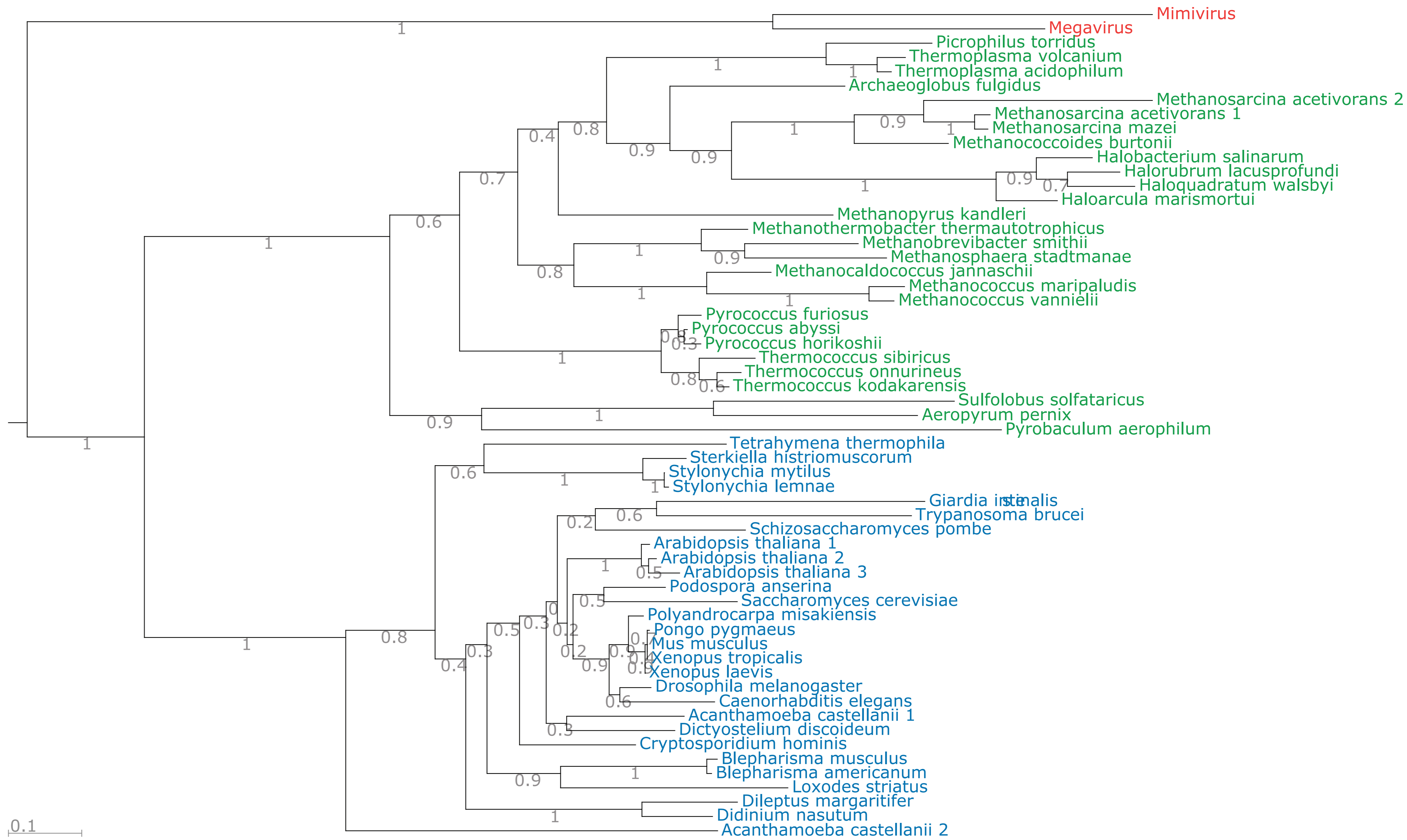

Supplement: Figure S8 — Phylogeny of R726, mg280 and cellular class-I RFs. A) The phylogenetic tree was built using MrBayes. Mimivirus and Megavirus sequences are shown in red, archaeal sequences in green and eukaryotic sequences in blue. Branch support shown represents posterior probability and bar represents 0.1 substitutions per site. B) Phylogenetic tree with the same sequences using PhyML. The bootstrap values from 100 replicates are shown (ranging from 0 to 1). The bar represents 0.1 substitutions per site. (PDF) [file pgen.1003122.s008.pdf]

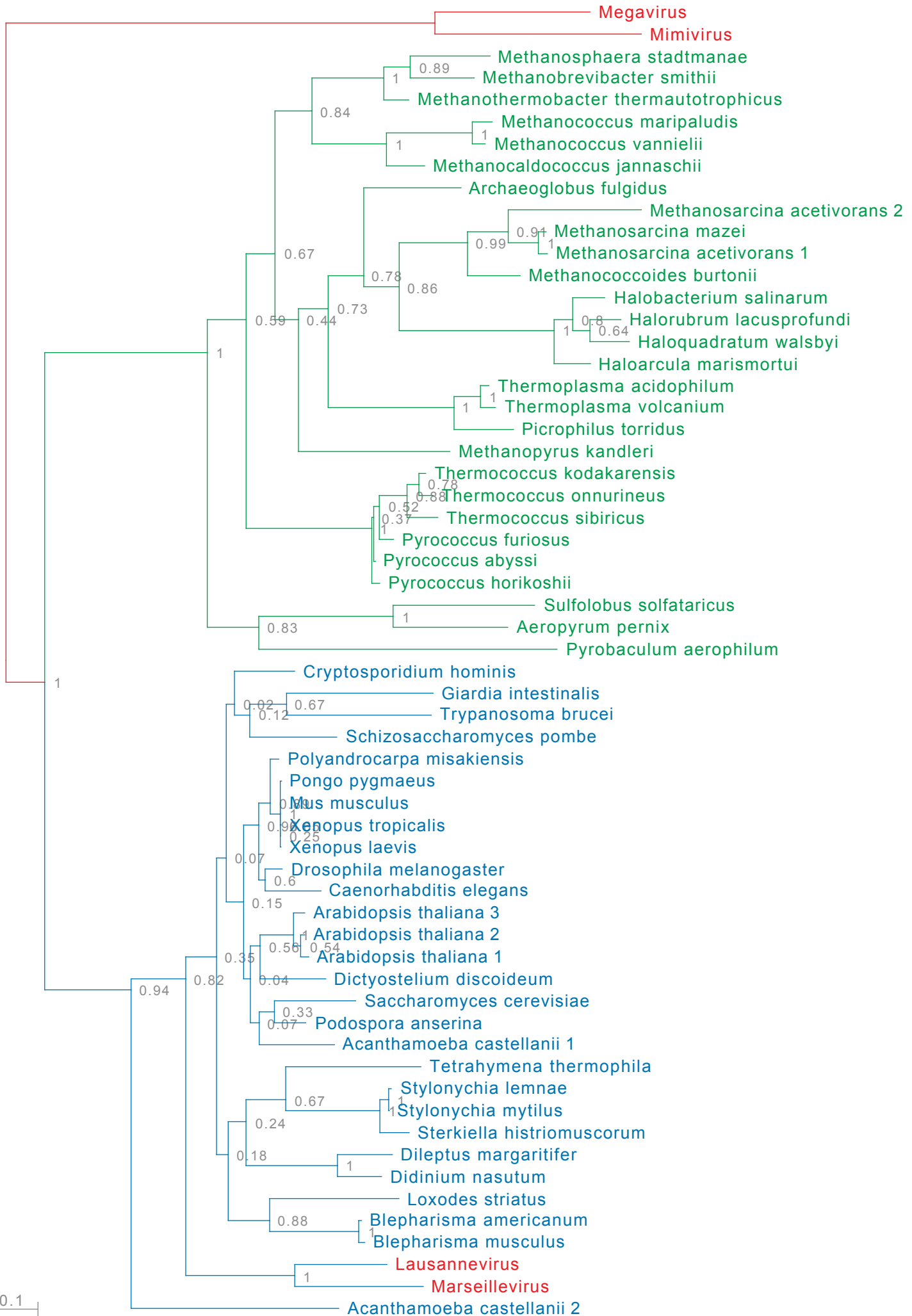

Supplement: Figure S9 — Phylogeny of R726, mg280, Marseillevirus, Lausannevirus and cellular class-I RFs using PhyML. The bootstrap values from 100 replicates are shown (ranging from 0 to 1). Viral sequences are shown in red, archaeal sequences in green and eukaryotic sequences in blue. Branch support shown represents posterior probability and the bar represents 0.1 substitutions per site. (PDF) [file pgen.1003122.s009.pdf]

A

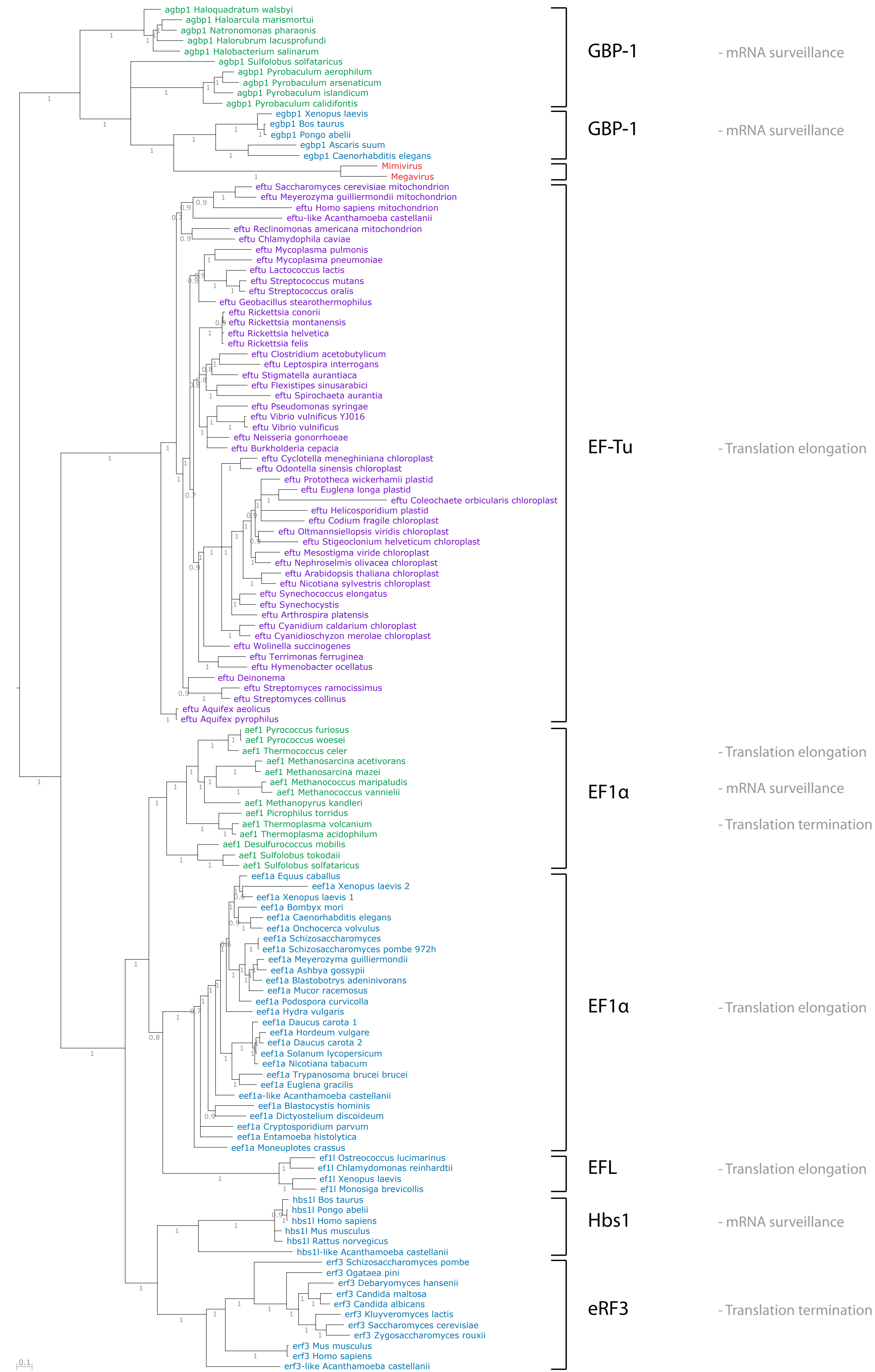

B

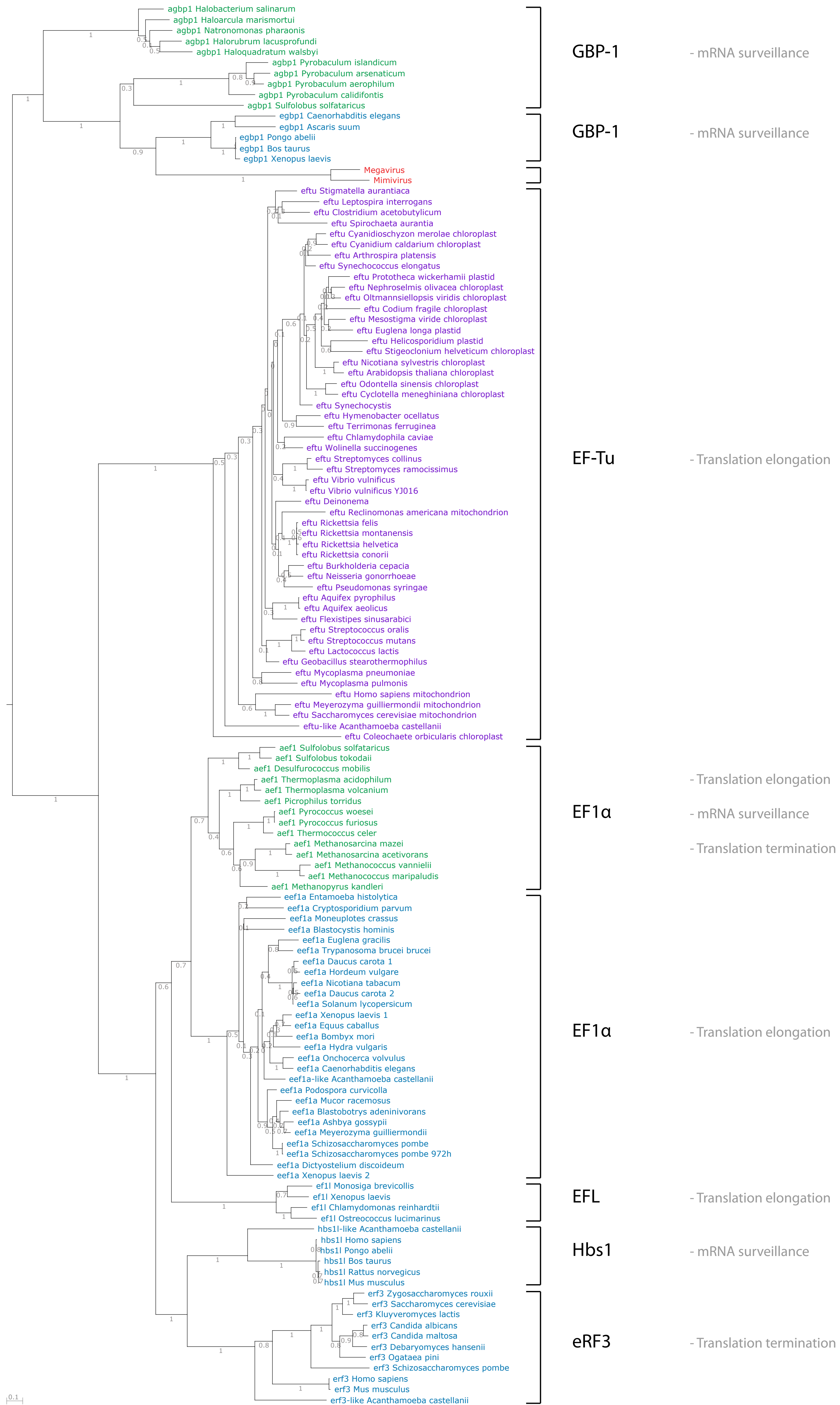

Supplement: Figure S10 — Phylogeny of the Mimivirus GTPase (R624), the Megavirus GTPase (mg752) and other cellular translational GTPases. The phylogenetic trees were built using MrBayes (A) and PhyML (B). Mimivirus and Megavirus sequences are shown in red, archaeal sequences in green, eukaryotic sequences in blue and bacterial sequences in purple. Each clade represents a translational GTPase subfamily. Associated functions are also shown. The bar represents 0.1 substitutions per site. (PDF) [file pgen.1003122.s010.pdf]
