# Supplementary material for: Translation in Giant Viruses: A Unique Mixture of Bacterial and Eukaryotic Termination Schemes
Source: PLoS Genet. 2012 Dec 13;8(12):e1003122. doi: 10.1371/journal.pgen.1003122 (PMC3521657; doi:10.1371/journal.pgen.1003122)
Supplement: Figure S7 — Identification by mass spectrometry of the full-length R726 protein expressed from the R726 RT mutant construct. The full-length R726 protein was identified with an E-value of 9.4e−17. In red are shown the trypsin digested peptides matching the sequence. (PDF) [file pgen.1003122.s007.pdf]

R726_SUMO **Mass:** 57151 **Expect:** 9.4e-17

1 M**GSSHHHHHH SPRMSDSEVN QEAKPEVKPE VKPETHINLK VSDGSSEIFF**

51 **K**IKKTTPLRR **LMEAFAKRQG KEMDSLRFLY DGIRIQADQT PEDLDMEDND**

101 **IIEAHREQIG PR**LEVLFQGP GSEFELITDP ELLQNLTQAI SQINSTDLVT

151 LYVPANNNLW LFVEHINKEL STAPNIKNKQ LR**KSVIQNLQ TINYQLK**TIK

201 **NIPETGLVVC AGNYTIKK**TH TTNSREILSC LSK**NYHVFVP PNPVVK**FYYK

251 CDK**KFHLEDL IKLYNDNGKK YAIVLISGKV TYFYQYTVNN TK**LLK**TITQD**

301 **IPNKHKTGGQ SAQRFER**IRD GSIKQYSKKI LEIMIQLYTS NGNFDYTCLI

351 LAGPAEMK**NI VIDHDLFSIF K**DHVSCIHNI SEITDNSISQ VVSMSIESID

401 SDNIDSIKEF ENKLQNPIKT NLFVFGSK**IV LKLFGLNRLS DIYITSDYYD**

451 **IETILENRGK CSVHYMDPIL FKKYGDIVGV KYYEYDYNDE YDYNY**
