# Supplementary material for: Translation in Giant Viruses: A Unique Mixture of Bacterial and Eukaryotic Termination Schemes
Source: PLoS Genet. 2012 Dec 13;8(12):e1003122. doi: 10.1371/journal.pgen.1003122 (PMC3521657; doi:10.1371/journal.pgen.1003122)
Supplement: Table S1 — Translation termination factors. (PDF) [file pgen.1003122.s011.pdf]

Table S1

	Bacteria	Eukaryotes	Archaea	Giant Viruses
Class-I Release Factors	RF1 RF2	eRF1	aRF1	R726/mg280
Recognized Stop Codons	↙ ↘↙ ↘<br>UAG UAA UGA	↙ ↓ ↘<br>UAG UAA UGA	↙ ↓ ↘<br>UAG UAA UGA	↙ ↓ ↘<br>UAG UAA UGA
Recoding Events	Frameshift in RF2	None	None	Frameshift + Readthrough
Class-II Release Factors	RF3 or no class-II RF	eRF3	aEF1α	Not known
