# Supplementary material for: Translation in Giant Viruses: A Unique Mixture of Bacterial and Eukaryotic Termination Schemes
Source: PLoS Genet. 2012 Dec 13;8(12):e1003122. doi: 10.1371/journal.pgen.1003122 (PMC3521657; doi:10.1371/journal.pgen.1003122)
Supplement: Table S2 — Selenocysteine incorporation protein machinery in A) A. castellanii and B) Mimivirus. (PDF) [file pgen.1003122.s012.pdf]

Table S2

A

Protein profile	HMMer E-value	HMMer Match	SECIS element (SECISearch)	STOP codon
Sec-tRNA synthase	7.2E-192	g14143	No	UAG
L-seryl tRNA-sec kinase	1.2E-38	g461	No	UGA
SECIS Binding Protein 2	4.7E-51	g1268	No	UAA
Sec-specific translation elongation factor	2.2E-167	g4522	No	UAG
tRNA-Sec associated protein	2.4E-115	g979	No	UGA

B

Protein profile	HMMer E-value	HMMer Match	SECIS element (SECISearch)	STOP codon
L-seryl tRNA-sec kinase	5.7E-06	R277	No	UAA
Sec-specific translation elongation factor	1.7E-23	R624	No	UAA
