# Supplementary material for: Translation in Giant Viruses: A Unique Mixture of Bacterial and Eukaryotic Termination Schemes
Source: PLoS Genet. 2012 Dec 13;8(12):e1003122. doi: 10.1371/journal.pgen.1003122 (PMC3521657; doi:10.1371/journal.pgen.1003122)
Supplement: Table S3 — Putative selenoproteome in A) A. castellanii and B) Mimivirus. (PDF) [file pgen.1003122.s013.pdf]

Table S3

A

Protein profile	HMMer E-value	HMMer Match	SECIS element (SECISearch)	STOP codon
Thioredoxin reductase	3.40E-253	g7770	Yes	UGA
Selenoprotein O	5.60E-221	g11791	Yes	UGA
Deiodinase iodothyronine	3.00E-17	g10588	Yes	UGA
15 kDa selenoprotein	2.10E-07	g7985	Yes	UGA
Selenoprotein J	2.90E-66	g8841	No	UGA
Glutathione peroxidase	3.40E-63	g9125	No	UAA
EhSEP2	3.90E-63	g295	No	UAA
FrnE	6.00E-60	g7250	No	UAG
Selenoprotein R	2.70E-56	g8784	No	UAG
Peptide methionine sulfoxide reductase	1.90E-55	g13684	No	UGA
Selenoprotein I	8.30E-50	g1732	No	UAG
Ostsp2	2.60E-34	g943	No	UAA
SelK	1.30E-13	g5423	No	UAG
Ostsp1	1.50E-09	g2445	No	UAG
Selenoprotein H	2.30E-06	g13126	No	UAG
Selenoprotein P	5.40E-06	g9757	No	UGA
Sel4	1.70E-03	g8666	No	UGA
SelTryp	4.70E-03	g7299	No	UGA

B

Protein profile	HMMer E-value	HMMer Match	SECIS element (SECISearch)	STOP codon
Thioredoxin	5.80E-17	R362	No	UAA
EhSEP2	3.30E-13	R362	No	UAA
Glutaredoxin	1.90E-11	R195	No	UAA
Selenoprotein J	2.40E-08	L543	No	UGA
Hdra	1.80E-07	R656	No	UAG
Prx_3	6.70E-07	R548	No	UAA
Methyl-viologen-reducing hydrogenase	7.30E-06	R656	No	UAG
Thiol:disulfide interchange protein	9.60E-05	R443	No	UAA
Peroxiredoxin	3.00E-04	R362	No	UAA
DSBA oxidoreductase	1.40E-03	R362	No	UAA
Methionine sulfoxide reductase A	2.20E-03	R901	No	UAA
Selenoprotein R	2.50E-03	L223	No	UAA
Ferredoxin-thioredoxin reductase	8.00E-03	L115	No	UAG
